# Miniaturized 3D Magnetic Force Sensor via Laser‐Assisted Folding and Magnetization for Enhanced Robotic Dexterity

**DOI:** 10.1002/advs.202524321

**Published:** 2026-03-27

**Authors:** Yujie Huang, Huangzhe Dai, Chengqian Zhang, Daofan Tang, Xinxin Zhang, Haonan Sun, Annan Ding, Xuebin Ni, Yizhi Zhang, Chengfeng Pan, Peng Zhao

**Affiliations:** ^1^ State Key Laboratory of Fluid Power and Mechatronic Systems, College of Mechanical Engineering Zhejiang University Hangzhou China; ^2^ Zhejiang Key Laboratory of Additive Manufacturing Technology and Equipment, School of Mechanical Engineering Zhejiang University Hangzhou China

**Keywords:** 3D force, laser‐assisted folding, magnetic force sensors, robotic manipulation

## Abstract

Magnetic tactile sensor with centripetally magnetization designs is capable of efficient 3D force decoupling sensing, which is essential for advancing robotic dexterity. Nevertheless, the miniaturization of sensors remains a challenge, primarily due to the complexities associated with precisely fabricating such planar magnetic structures. Here, we present a laser‐assisted folding and magnetization (LAFM) method to create centripetally magnetized films. Laser‐etched grooves enable controlled folding, achieving accurate magnetization alignment in films as small as 5 × 5 mm^2^, which are verified by root mean square errors (RMSEs) of less than 5 µT between the experimental and theoretical magnetic field values. This breakthrough enabled the compact 3D force sensor featuring high force resolution (tangential 3 mN, normal 9 mN), rapid response (34 ms), and long‐term stability (>2500 cycles, <1% deviation). When installed on a mobile manipulator, the sensor enables adaptive grasping of delicate objects during obstacle traversal. Its functionality is further enhanced by deploying an array of 16 units on a dexterous hand, which supports non‐destructive stiffness recognition across six representative materials and stable manipulation of variable‐mass or irregular objects. This work establishes a robust pathway for miniaturized tactile sensors and embodied intelligence, especially in robotic perception.

## Graphical abstract

To achieve 3D force sensing in confined spaces such as fingertips, we developed a laser‐assisted folding and magnetization method to fabricate miniature films with precise centripetal magnetization. The resulting compact sensor delivers high force resolution, rapid response, and long‐term stability, significantly enhancing the manipulation capabilities of mobile robots and dexterous hands.

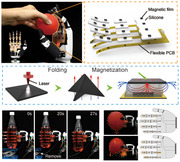

## 1 Introduction

With the increasing sophistication of artificial intelligence foundation models and the growing capability of robotic hardware, robotics is evolving from pre‐programmed automation toward embodied intelligence [1, 2]. Dexterous robotic hands, serving as a key medium for interactions between robots and environments, are expected to replicate the form and motion of human hands [3, 4, 5, 6, 7]. Tactile sensors are of critical importance and charm, attracting significant research on integrating human‐like perceptual capabilities in dexterous hands [8, 9, 10]. By obtaining tactile information, robots can identify the physical properties of objects (e.g., texture, stiffness) and monitor gripping forces. Extensive research efforts have focused on developing tactile sensors with high sensitivity [11, 12, 13], wider detection range [14, 15, 16, 17, 18], and multimodal sensing capabilities [19, 20, 21, 22, 23]. In dynamically changing or unstructured environments, robots require more comprehensive force information to interpret and respond to contact interactions. Against this background, 3D force sensing has emerged as an important research direction [15, 24, 25, 26, 27]. Specifically, normal force information is essential for determining contact state and magnitude, while shear force sensing facilitates slip detection and compensation for external disturbances. The integration and processing of 3D force information can significantly strengthen the stability and reliability of robotic manipulation in complex operational scenarios.

However, effectively decoupling normal and shear forces within flexible substrates to achieve high‐resolution 3D force sensing remains a significant challenge. Researchers have explored various physical mechanisms for 3D force sensors, including resistive [28, 29, 30, 31], capacitive [32, 33, 34], triboelectric [35, 36], and optical mechanisms [37, 38, 39]. However, strategies such as designing 3D microstructures and integrating sensor array often involve complex fabrication processes and high cost. In contrast, benefiting from the vector characteristic of the magnetic field, magnetic tactile sensors offer an efficient solution [20, 24, 40, 41, 42, 43, 44]. By combining innovative self‐decoupling theory with strategic magnetization designs, magnetic tactile sensors can achieve high‐precision 3D force perception using solely a single Hall element [41]. This approach enables simplified structures, reduced computational complexity, and enhanced generalization, making magnetic tactile sensors outstanding for human‐like perception in dexterous hands, particularly in fingertips, where stringent size constraints exist.

The accurate reconstruction of 3D forces from magnetic signals critically depends on the precise fabrication of the magnetic structures with centripetal distribution patterns. This requirement becomes challenging during miniaturization. Current fabrication techniques for magnetic structure primarily include magnetic field‐assisted 3D printing [45, 46, 47, 48], adhesive assembly [49], and folded magnetization [41, 50]. Magnetic field‐assisted 3D printing enables the fabrication of spatial 3D magnetization [45, 47], but faces limitations in magnetic particle loading density [51, 52] and external field strength, often resulting in insufficient magnetic field intensity. Adhesive assembly [49] offers fabrication simplicity, but struggles to achieve high‐precision magnetic arrangement. Folding‐based magnetization and other special magnetization methods (such as curling, overlaying, and splicing) hold potential for producing complex planar magnetization. However, at miniature scales, inherent material stiffness restricts precise and controllable operation, thus limiting the force accuracy and structural consistency of sensors (See Table S1 for more details).

Here, we propose a laser‐assisted folding and magnetization (LAFM) process to fabricate small‐scale magnetized films with precise centripetal magnetization. This technique utilizes laser processing to create groove structures on the film surface, reducing the local stiffness of the material. Assisted by specialized fixtures, the film undergoes controlled folding and then magnetized under a high‐intensity external magnetic field. Using this method, we fabricated centripetally magnetized films with precise magnetic arrangements, demonstrating areas from 400 mm^2^ down to 25 mm^2^. Both simulations and experiments confirmed the effectiveness of the LAFM process. Simulation results indicated that while the groove structures slightly reduce the magnetic field strength of the film, this effect can be compensated for by introducing a correction coefficient. Experiments demonstrated that a 5 × 5 mm^2^ film achieved a magnetic field distribution highly consistent with the theoretical model (tangential RMSE < 2 µT, normal RMSE < 5 µT). We further developed a 3D force sensor that delivers high sensitivity (tangential 3 mN, normal 9 mN), rapid response (34 ms), and excellent stability (>2500 cycles, <1% deviation). Integrated into a mobile manipulator, the sensor enabled adaptive grasping of a cherry tomato during obstacle crossing by detecting shear force changes. When mounted on a dexterous hand with 16 sensors, the system performed non‐destructive stiffness identification on six different material types and achieved stable grasping of variable‐mass bottles and irregular objects like pinecones and gamepad. In summary, the LAFM process enables precise centripetal magnetization of miniaturized films, paving the way for 3D force sensors with high sensitivity and fast responsibility. This advancement boosts the dexterity, stability of robots operating in complex environments.

## 2 Results

### 2.1 Laser‐Assisted Folding and Magnetization Process for Miniaturized 3D Magnetic Tactile Sensors

**FIGURE 1:**
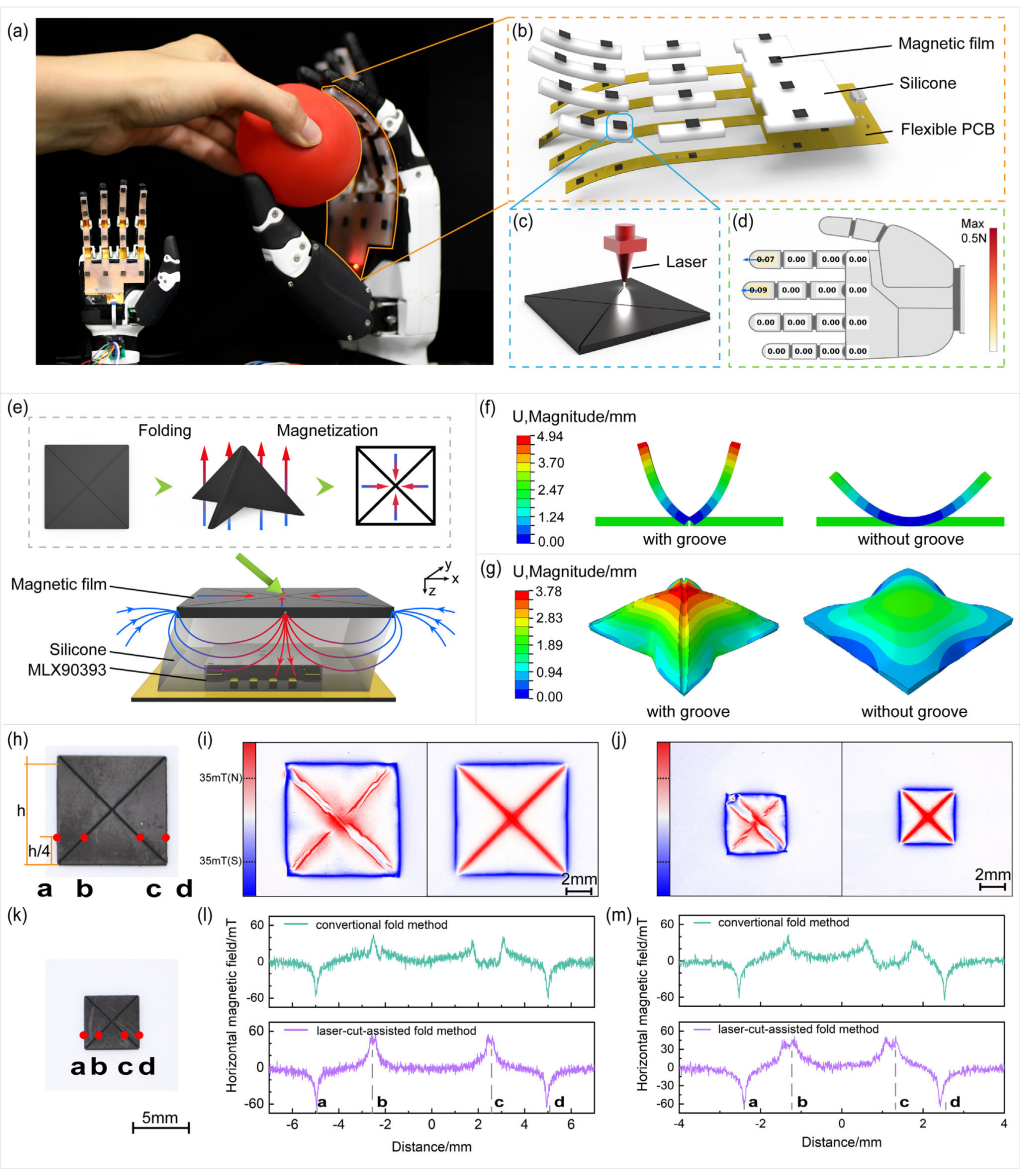
The LAFM process to fabricate miniaturized 3D force tactile sensors with precise centripetally magnetized films. (a) Sensor integration and system overview. (b) Exploded view of the sandwich‐structured sensors. (c) Laser etching of grooves on the magnetic film. (d) GUI for real‐time 3D force visualization. (e) Sensing mechanism under external forces. (f) FEA deformation for grooved and non‐grooved beams. (g) FEA deformation for grooved and non‐grooved magnetic films. (h) The LAFM‐fabricated magnetic film (10 mm side length). (i) Magneto‐optic images of magnetic domains from conventional and LAFM methods (10 mm side length). (j) Magneto‐optic images of magnetic domains from conventional and LAFM methods (5 mm side length). (k) The LAFM‐fabricated magnetic film (5 mm side length). (l) Magnetic field profiles at one‐quarter side length (10 mm side length). (m) Magnetic field profiles at one‐quarter side length (5 mm side length).

The advancement of robotic intelligence creates a need for human‐level dexterous manipulation, a capability that depends heavily on the performance of tactile sensors. While sensors are required in areas like the fingertips for complex hand functions, the spatial constraints place needs on their size (Figure 1a). These challenges necessitate tactile sensors that simultaneously deliver structural simplicity and high precision. Magnetic tactile sensors, owing to their self‐decoupling capability, have emerged as a promising solution. This decoupling mechanism is derived from an idealized infinitely periodic sinusoidal magnetization pattern and self‐decoupling theory. While true infinite periodicity is physically unachievable, previous studies have confirmed that finite‐period magnetic films can approximate ideal magnetization behavior, after linear correction of the magnetic flux density along the z direction (*B_z_*), easing the fabrication of magnetization [41]. Nonetheless, realizing small‐scale magnetic structures remains a critical challenge, where fabrication precision directly impacts decoupling accuracy and sensitivity. To address this challenge, we propose the LAFM process, which enables the construction of a small‐scale magnetic tactile sensor with 3D force decoupling capability.

As shown in Figure 1b, the proposed LAFM 3D force sensor consists of three functional layers from top to bottom, including centripetally magnetized film units, a stiffness‐tunable elastomer, and a printed circuit board (PCB) whose core components are Hall sensors (MLX90393). The elastomer was manufactured using Ecoflex 00–30 as the base material. Its hardness can be modulated by incorporating silicone oil or polydimethylsiloxane (PDMS). The sensing mechanism operates as follows: external forces deform the elastomer, causing displacement of the magnetic film and thereby altering the spatial magnetic field distribution, as illustrated in Figure 1e. These variations are captured in real‐time by a Hall sensor. Subsequently, 3D decoupling algorithms process the signals to compute the decoupling coefficients (*R_x_*, *R_y_*, and *S_z_*). The 3D force (*F_x_*, *F_y_*, *F_z_*) vector is determined via pre‐calibrated models that establish the relationships among the decoupling parameters, film's displacement, and applied external force. The resolved force vectors are visualized through a graphical user interface (GUI, Figure 1d), where tangential forces (*F_x_*, *F_y_*) are represented by directional arrows, and the normal force (*F_z_*) magnitude is simultaneously displayed using a numerical readout and a color gradient. The decoupling calculation formulas are presented as follows:

(1)

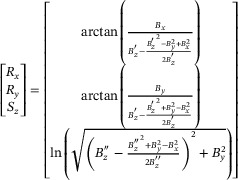

where *B_x_* and *B_y_* represent the magnetic flux density along the *x* and *y* directions, respectively. 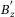 and 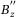 denote the corrected *B_z_* signals, processed through a linear calibration function to ensure better consistency with the theoretical model.

Central to the sensor's fabrication is the LAFM process (Figure 1c; Figure S1), where a CO_2_ laser (Speedy 300, Trotec Laser GmbH) is employed to precisely etch grooves onto a magnetic film. This subtractive technique selectively reduces local stiffness, thereby enhancing the film's capacity for localized deformation. Finite element analysis (FEA) validates this design (simulation parameters provided in Table S2), demonstrating that grooved structures exhibit superior deformability under load compared to non‐grooved counterparts (Figure 1f,g). Since laser processing parameters directly dictate the critical dimensions (depth and width) of these grooves, we established a quantitative mapping between the processing inputs and the resulting groove morphology. As shown in Figure S2, increasing laser power or decreasing scanning speed yields wider and deeper grooves, consistent with the energy density principle. To determine the optimal processing parameters, we combined these fabrication trends with FEA simulations. The simulations revealed that when the groove depth reaches 60%‐80% of the film thickness, the localized stiffness decreases significantly (Figure S3). Furthermore, the groove width exhibits a threshold effect whereby the introduction of even a minimal 20 µm width triggers a sharp 161% increase in deformability. These findings indicate that the etched grooves effectively break the material's mechanical continuity, providing an essential path for stress relief during folding. Consequently, for the 0.5 mm thick magnetic film, a laser power of 15 W (30% of maximum) and a scanning speed of 71 mm/s (2% of maximum) were selected. To verify process consistency, we conducted a repeatability study by measuring multiple groove sections across different samples. The results showed high uniformity, with standard deviations of 6.14 µm in depth and 1.83 µm in width (Figure S4). This dimensional precision enables reliable folding, which is ensured with the aid of customized 3D‐printed fixtures. The folded structure is then exposed to a 4 T magnetic field to achieve the final centripetal magnetization. Finally, to evaluate the impact of these structural features on magnetic performance, simulations were conducted (Figure S5 and Table S3). The field was sampled on two planes (0.5 mm and 0.425 mm below the film) along the X‐axis. Comparison of models with and without grooves confirms that the grooves cause a proportional scaling of all field components without distorting the field's fundamental spatial configuration. To quantify this effect, we applied a unified correction coefficient *a_0_* (1.04, obtained via fitting). After correction, the RMSEs of the magnetic field components (*B_x_*, *B_y_*, *B_z_*) were reduced to 2.29, 1.58, and 2.42 µT at the 0.5 mm plane, and to 4.70, 3.34, and 4.47 µT at the 0.425 mm plane. A single coefficient *a_0_*, thus effectively compensates for the global, proportional influence of the grooves. Critically, the employed 3D force decoupling algorithm is inherently robust to such uniform scaling. In its core equations, each field component appears with identical order in both the numerator and denominator (Equation 1). Consequently, any unified proportional coefficient cancels out mathematically, leaving the final decoupled force parameters unchanged.

**FIGURE 2:**
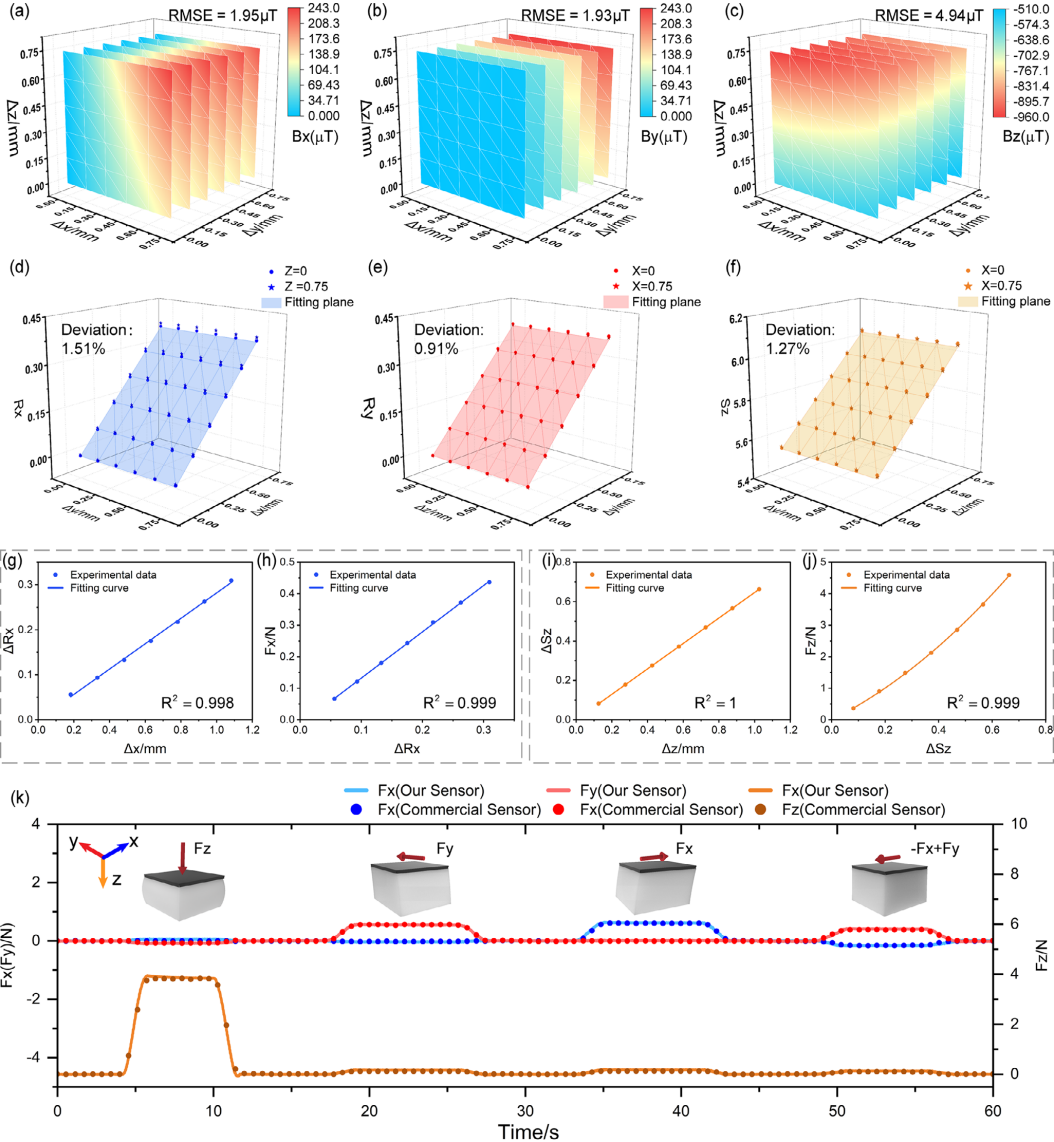
3D force decoupling properties of miniaturized magnetic tactile sensors. (a) The measured magnetic field components *B_x_*. (b) The measured magnetic field components *B_y_*. (c) The measured magnetic field components *B_z_*. (d) Evaluation of self‐decoupling performance via spatial mapping of coefficients *R_x_* for the 5 mm unit. (e) Evaluation of self‐decoupling performance via spatial mapping of coefficients *R_y_* for the 5 mm unit. (f) Evaluation of self‐decoupling performance via spatial mapping of coefficients *R_z_* for the 5 mm unit. (g) Linear relationships between *∆R_x_* and *∆x*. (h) Linear relationship between *F_x_* and *R_x_*. (i) Linear relationships between *∆S_z_* and*∆z*. (j) Second‐order polynomial relationship between *F_z_* and *S_z_*. (k) Force tracking performance under sequential multi‐axis loading.

The efficacy of laser‐assisted folding in controlling magnetic domains was compared against a traditional approach (knife‐cutting of external contours followed by folding and magnetization). As shown in Figure 1i,j, magneto‐optic imaging (MagView, Matesy GmbH, Germany) revealed that the laser‐assisted method produced a highly uniform magnetic domain distribution along the folds, closely matching the ideal centripetal distribution with an orthogonal sinusoidal period (1 × 1). Quantitative analysis further demonstrated the superiority of the laser‐assisted technique. For the square magnetic film samples with side lengths of 10 mm and 5 mm (Figure 1h,k, respectively), the measured peak magnetic field at one‐quarter height deviated by less than 1.7% and 4% from theoretical predictions. In contrast, the traditional folding method exhibited multiple magnetic field distortion peaks in the same region (Figure 1l,m). Furthermore, the spatial magnetic field distribution beneath the magnetic film was characterized, and the experimental results were compared with the finite element simulation data. Quantitative validation via spatial magnetic field measurements beneath the film (*B_x_*, *B_y_*, and *B_z_*) shows close alignment with theoretical models (Figure 2a–c; Figure S6). The root‐mean‐square errors (RMSEs) for *B_x_*, *B_y_*, and *B_z_* are 1.95, 1.93, and 4.94 µT, respectively. These findings confirm the precise magnetization control achieved by the proposed process.

### 2.2 Sensing Properties of Miniaturized 3D Magnetic Tactile Sensors

The effectiveness of self‐decoupling is pivotal for the 3D force precision of tactile sensors. According to the decoupling model, the displacement and the decoupling coefficients are linearly related by the expressions:

(2) $$R_{x}=\frac{1}{b}\;\cdot x,\;R_{y}=\frac{1}{b}\;\cdot y,\;S_{z}=\frac{1}{b}\;\cdot z$$

where *b* is a constant related to the side length of the magnetic film. Consequently, in the ideal case, the response surface constructed from *x, y, R_x_* in 3D space should form a linearly inclined plane.

The impact of scaling on self‐decoupling performance was evaluated experimentally in magnetized films with side lengths of 5, 10, 15, and 20 mm (Figure 2d–e; Figure S7). The miniaturized 5 × 5 mm^2^ unit was analyzed in detail. We performed sampling across the region *x*, *y* ∈ [0,0.75] mm at a resolution of 0.15 mm over two distinct height planes (Z = 0 mm and Z = 0.75 mm). This yielded two sets of 6 × 6 experimental data matrices for *R_x_* (Figure 2d). Compared with the ideally decoupled model, the average relative error of *R_x_* was 1.51%. Using the same methodology, experimental data for *R_y_* and *S_z_* were acquired on two longitudinal sections (X = 0 mm and X = 0.75 mm), as shown in Figure 2e,f. The corresponding average relative errors were calculated as 0.91% and 1.27%, respectively. These results demonstrate that the fabricated magnetic film unit maintains excellent 3D displacement decoupling capability, even at a compact scale, achieving sub‐millimeter displacement detection with a resolution as high as 0.01 mm.

Accurate 3D force sensing was achieved by establishing a quantitative mapping between the decoupling coefficients and the applied forces. The magnetic film was integrated with an elastomer substrate for quasi‐static compression tests along the *x*, *y*, and *z* axes. The multi‐axis calibration platform, incorporating a commercial six‐axis force sensor (KWR63B, KUNWEI, China) as a reference, was constructed to correlate the decoupling outputs with the applied forces. The results indicated a strong linear relationship (*R^2^* ≥ 0.998) between the decoupling coefficients and displacement in all directions (Figure 2g,i). For forces sensing, *F_x_* exhibited a linear relationship with their corresponding displacements (and thus with *R_x_* or *R_y_*), as shown in Figure 2h. In contrast, *F_z_* required a second‐order polynomial fit due to the hyperelastic nature of the elastomer substrate, relating *S_z_* to *F_z_* (Figure 2j). The sensor, with compact dimensions of 5 × 5 × 3 mm^3^, was fabricated using a silicone elastomer composite (Ecoflex 00–30: PDMS = 1:1 by mass). It demonstrated reliable operation across 0–4.5 N (normal) and 0‐0.45 N (tangential) force ranges, with detection resolutions of 3 and 9 mN, respectively. Finally, dynamic tests were conducted on the calibrated sensor system. Over 60 s, forces were sequentially applied along the *z*, *y*, *x*, and *x*‐*y* directions (Figure 2k). The sensor outputs were compared against the reference readings from the KWR63B. The mean absolute errors (MAEs) in the *F_x_*, *F_y_*, and *F_z_* directions were 0.016 N, 0.015 N, and 0.028 N, respectively, further demonstrating the system's high accuracy in multi‐axis dynamic force sensing applications.

**FIGURE 3:**
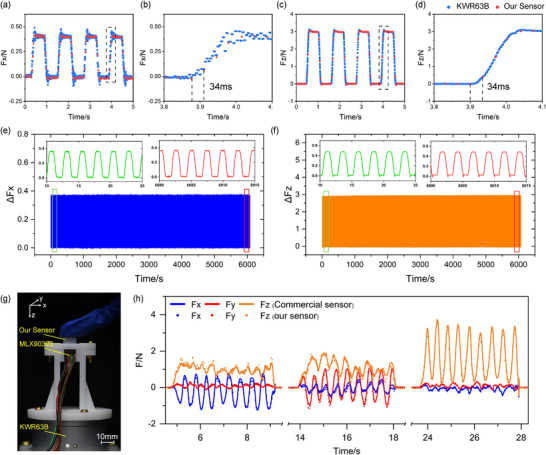
Dynamic response characterization, cyclic durability, and real‐word interaction performance of the sensor. (a) The *F_x_* signal measured by our sensor closely tracks the signal from the KWR63B reference. (b) Response time of the *F_x_* component. (c) The *F_z_* signal measured by our sensor matches the KWR63B reference signal. (d) Response time of the *F_z_* component. (e) Variation of the *F_x_* output over 2500 loading/unloading cycles. (f) Variation of the *F_z_* output over 2500 loading/unloading cycles. (g) Experimental setup for finger pressing. (h) Comparison of the 3D force output (*F_x_*, *F_y_*, *F_z_*) between our sensor and the KWR63B during random finger pressing.

The dynamic response of the magnetic 3D force sensor was quantified by measuring output signal decay time under rapidly changing external loads. Using KWR63B (sampling frequency: 1000 Hz) as the reference, output signals from both sensors were compared within identical time windows. As demonstrated in Figure 3a,c, the magnetic sensor outputs closely track the KWR63B signals with negligible phase lag. The measured dynamic response time is about 34 ms (Figure 3b,d), which is on par with the tactile response speed of human skin. This rapid responsiveness supports low‐latency 3D force data acquisition, making the sensor well‐suited for fast interaction and real‐time feedback applications. Sensor stability and lifespan were assessed through periodic compressive loading in shear and normal directions, applied via a 3D motion platform (PMS‐3). Over a continuous 6086 s testing period, the sensor completed 2500 loading/unloading cycles. Throughout the entire test, the output amplitudes of *F_x_* and *F_z_* remained highly consistent (Figure 3e,f). A comparison between the output signals at the beginning and end of the test (each within a 15 s window) showed that force deviation was consistently maintained within 1%. These results strongly confirm the sensor's outstanding long‐term stability and durability. To assess humidity robustness, the sensor was fully immersed in water for 1 h. Compared to baseline readings in air, the deviation was negligible. A slight output increase was observed, which is attributed to hydrostatic pressure acting on the sensor structure rather than humidity effects. Even considering this pressure‐induced shift, the overall average deviation remained below 3  µT (Figure S8), confirming the sensor's reliability in high‐humidity and underwater conditions. Real‐world interaction performance was further evaluated through finger‐pressing experiments, as shown in Figure 3g. Testers applied periodic pressure sequentially along the *x*, *y*, and *z* directions on the sensor surface while synchronously recording the output data from both the magnetic sensor and the KWR63B. During the 33 s dynamic operation process, the collected 3D force data was comparatively analyzed (Figure 3h; Movie S1). The MAEs for *F_x_*, *F_y_*, and *F_z_* forces were 0.039 N, 0.029 N, and 0.086 N, respectively. These results indicate that this 3D magnetic tactile sensor not only operates stably but also tracks complex, non‐constant dynamic tactile input changes with high precision.

### 2.3 Adaptive Grasp under Disturbances

Achieving reliable object grasping in complex, unstructured environments, particularly when facing dynamic disturbances such as vibrations or varying payloads, presents the core challenge of balancing grasp stability with object integrity. This balance heavily depends on accurate real‐time force feedback and the capability for dynamic grip force modulation. 3D force sensors enable simultaneous measurement of both normal (*F_n_* = *F_z_* ) and tangential ($F_{s}=\sqrt{F_{x}^{2}+F_{y}^{2}}$) forces. This multi‐directional force information provides a richer physical interaction model, facilitating robust adaptive grasp control strategies.

**FIGURE 4:**
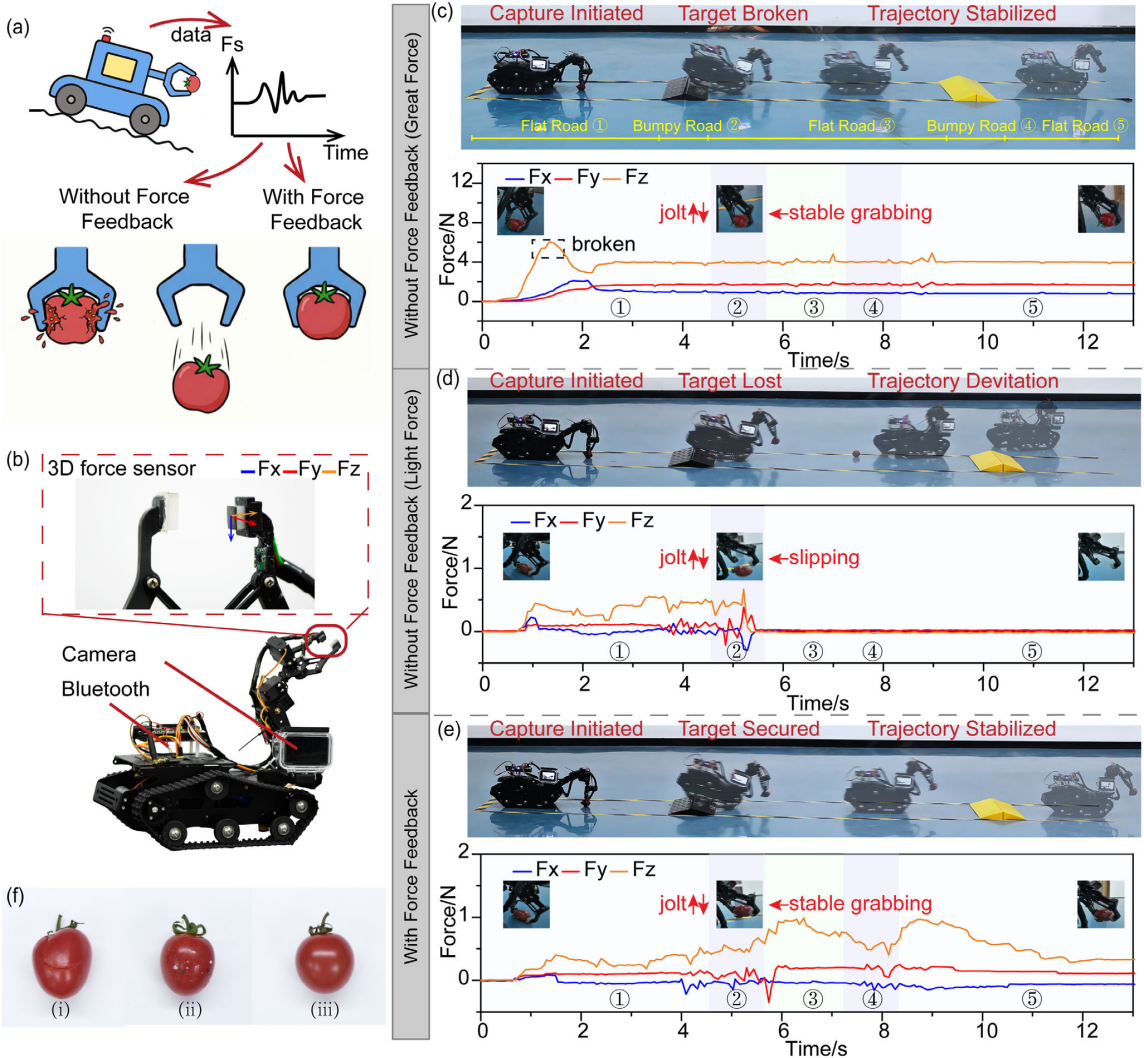
Obstacle‐crossing experiment comparing grasp performance. (a) Diagram of the control logic. (b) Experimental setup with the mobile platform, robotic arm, and a 3D force sensor. (c) Scenario I: high constant force (non‐adaptive). (d) Scenario II: low baseline force (non‐adaptive). (e) Scenario III: proposed adaptive strategy (with feedback). (f) Post‐experimental inspection of the cherry tomatoes from the three scenarios.

For objects with considerable mass, grasp stability under disturbances can be reliably assessed using the *F_s_*/*F_n_* ratio (*r*). For instance, when progressively filling a 250  mL plastic bottle from empty to 200  mL, stable grasping was maintained as long as the r stayed within 0.4–0.6 (Figure S9 and Movie S2). This was achieved through closed‐loop control, in which the gripping force was adaptively adjusted in response to changes in liquid weight, ensuring that the ratio *r* remained within the stability range. Applying the same closed‐loop strategy to a fragile egg (*r*: 0.2–0.3), the system demonstrated robust stability against multidirectional manual interference, preserving a safe hold via real‐time force adjustment (Figure S10 and Movie S2). However, lightweight objects present a distinct challenge. Inherently small *F_n_* and *F_s_* magnitudes mean even slight disturbances can cause large swings in the ratio *r*. As a result, *r* becomes noisy and unreliable as a slip indicator under dynamic conditions. In practice, we therefore adopt a hybrid control strategy. During the initial static phase, prior to disturbance, *r* is used to establish a stable baseline grip force that is sufficient for holding without over‐constraining the object. Once the object is lifted, the control strategy transitions to real‐time monitoring of *F_s_* to detect dynamic disturbances. The system continuously evaluates *F_s_* for abrupt or discontinuous variations that exceed a predefined dynamic threshold, indicative of potential slippage (Figure 4a). Upon detecting such an event, the controller adaptively increases the grip force within a bounded range to preserve grasp stability while minimizing the risk of mechanical damage to delicate objects. Furthermore, when *F_s_* fluctuations remain within acceptable limits, and the applied grip force exceeds the baseline, the system slightly reduces the gripping force to alleviate unnecessary load without compromising stability.

We validated this strategy through comparative obstacle‐crossing experiments (Movie S3) using a mobile robotic platform equipped with a 3D force sensor at the end‐effector, as shown in Figure 4b. To mitigate interference from environmental magnetic fields, an auxiliary Hall sensor was positioned adjacent to the force sensor. The task required the robot to transport a delicate cherry tomato across two slopes of varying gradients (Obstacle A: height 30 mm, slope 16.7°; Obstacle B: height 35 mm, slope 19.3°) to simulate operation on non‐stationary, uneven terrain. In the first scenario, a conventional high‐force, fixed‐displacement approach (approximately 6 N) was evaluated. While this excessive force successfully prevented slippage, it resulted in a total failure regarding object integrity. Post‐experimental inspection revealed significant mechanical crushing and tissue damage (Figure 4f–i). This structural collapse was further confirmed by the force data, which showed an abrupt drop from 6 N to 3 N as the tomato's stiffness failed (Figure 4c). This outcome underscores that simply increasing gripping force to ensure stability is insufficient for handling fragile objects.

To further explore the limitations of non‐adaptive control, we conducted two additional experiments where the initial grasp was established using the force ratio strategy (*r* ≈ 0.4, *F_n_* ≈ 0.3 N) to ensure a gentle hold. In the second scenario, the gripper's displacement was held fixed following the initial grasp. While the grasp remained stable during the initial static phase, the system failed at approximately 5 s during the transition from the ramp to flat ground. Due to the absence of a chassis suspension system, terrain‐induced shocks and inertial perturbations were transmitted directly to the end‐effector. Without real‐time feedback, the controller could not compensate for tangential force spikes. This resulted in the tomato slipping, which both contaminated the fruit and interfered with the robot's motion trajectory, ultimately leading to task failure (Figure 4d).

In contrast, the implementation of the proposed hybrid strategy, which incorporates real‐time tangential force (*F_s_*) feedback, demonstrated superior performance. At 4 s, *F_s_* showed fluctuations, with a subtle compensatory increase in gripping force (Figure 4e). Subsequently, the normal force remained relatively constant, as no significant tangential disturbances were encountered in the immediately following interval. Between 5 s and 6 s, sustained oscillations in *F_s_* were observed due to external disturbances. During this phase, the system incrementally increased the gripping force, subject to a dynamic adjustment cap of three times the baseline (0.3  N), thereby limiting the maximum force to under 1.2  N. Separate destructive compression tests confirmed that the mechanical damage threshold exceeds 5  N (Figure S11), ensuring our upper limit remains well within safe margins. After 6 s, as the platform returned to a steady state and *F_s_* fluctuations diminished, the controller gradually reduced the grip force, while ensuring it remained above the baseline to prevent unintended release. A similar adaptive response was observed during traversal of the second obstacle: a spike in *F_s_* upon descent at approximately 8  s triggered timely grip reinforcement, followed by a controlled relaxation once the system stabilized at 9  s. Post‐experimental inspection confirmed that the cherry tomato remained entirely intact, devoid of visible indentations or structural damage (Figure 4f(iii)). This outcome validated that real‐time 3D force feedback enables robust manipulation in dynamic environments without compromising object integrity. Ultimately, this approach improves the system's adaptability to complex and dynamic operational scenarios without increasing mechanical complexity.

Additionally, we conducted a continuous grasping experiment on five lightweight and fragile objects: a dried leaf, a piece of tofu, a strawberry, a grape, and a tomato (Figure S12; Movie S4). While the force‐ratio strategy works for anti‐disturbance grasping when applied to a specific material type, it cannot accommodate the wide friction variation across different objects and fails on extremely light ones, such as the dried leaf. Therefore, we adopted the *F_s_* detection strategy. When the normal force reached a preset threshold ( *F_n_* = 0.1 N) and lifting began, the system continuously monitored *F_s_* fluctuations to detect slip tendencies and adjusted the grip force accordingly. The force curves show a transient initial peak due to sensor deformation during the gripper's composite clamping motion (*t_0_*‐*t_1_*), which rapidly attenuated upon lifting and did not interfere with subsequent slip detection. All objects were grasped continuously and without damage, demonstrating its precise perception and adaptive control capability in unstructured environments.

### 2.4 A Dexterous Hand With Distributed 3D Force Sensing for Real‐Time Stiffness Perception and Adaptive Grasp Control

To achieve large‐area and fine‐grained force perception, we integrated sixteen 3D tactile sensors into the robotic hand. The four fingers (excluding the thumb) each carry three sensors, positioned at the fingertip, finger pad, and near the finger root, while the additional four sensors are evenly distributed across the palm area. The fingertip and finger‐pad sensors serve as the primary sensing units, capable of detecting object stiffness and precisely adjusting grasping force in response to dynamic disturbances. Finger‐root sensors enhance the fingers’ ability to conform to objects with complex geometries, and palm sensors determine the contact position of the object within the palm. Given the integration density of the array, the potential magnetic crosstalk between adjacent sensor units was systematically evaluated. In the current layout, the minimum center‐to‐center distance between adjacent units is 15 mm. Both simulations (Figure S13 and experimental validations (Movie S5) confirm that at this spacing, magnetic crosstalk causes less than 1.35% deviation in decoupling coefficients, allowing each sensor to be treated as functionally independent.

**FIGURE 5:**
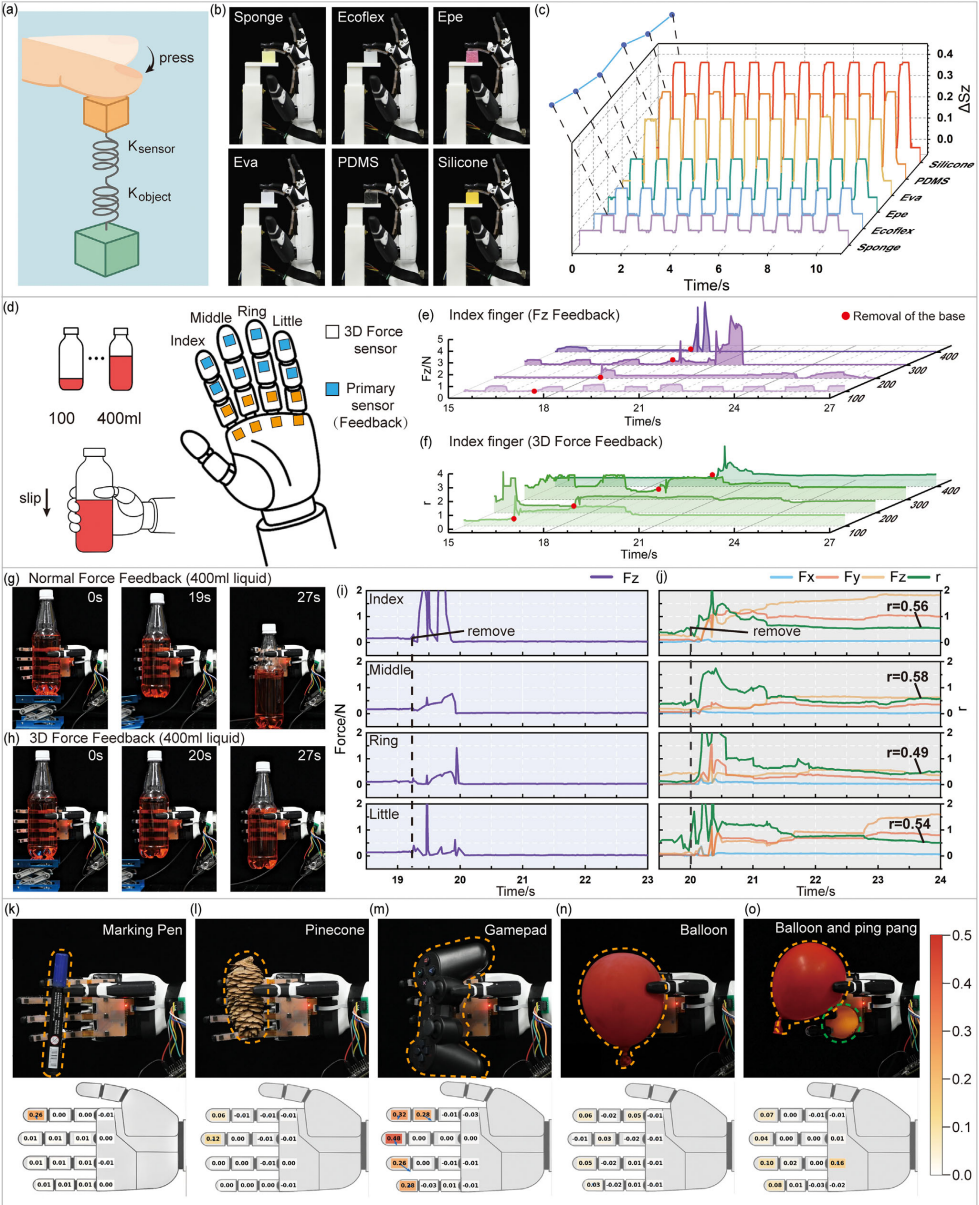
Stiffness perception and active grasping enabled by distributed 3D force sensors on a dexterous hand. (a) The contact mechanics of stiffness perception. (b) Six representative materials for stiffness identification. (c) *S_z_* response during active pressing. (d) Schematic of the grasp stability experiment and feedback principle. (e) *F_z_* of the index finger versus object mass (100‐400 mL). (f) Force ratio r of the index finger versus object mass (100‐400 mL). (g) Grasping process for the bottle with 400 mL liquid using *F_z_* feedback. (h) Grasping process for the bottle with 400 mL liquid using r feedback. (i) Sensor data under *F_z_* feedback (400 mL). (j) Sensor data under *r* feedback (400 mL). (k) Grasping a marker pen. (l) Grasping a pine cone. (m) Grasping a gamepad. (n) Grasping a balloon. (o) Sequential grasping of multiple objects.

Based on this sensor network, we performed non‐destructive stiffness identification. The contact mechanics principle can be modeled as two springs in series (Figure 5a): the senor spring (*k_1_*) and the object spring (*k_2_*) [29]. The overall system stiffness, defined as *k = ∆F_z_ / ∆z*, allows the object's stiffness *k_2_* to be derived as *k_2_ = k_1_k / (k_1_ – k)*. Under a fixed displacement *∆z*, both *∆F_z_* and *∆S_z_* exhibit a positive correlation with the *k_2_*. As shown in Figure 5b, we selected six representative flexible materials for validation: sponge, Ecoflex 00–30, expanded polyethylene (EPE), ethylene vinyl acetate (EVA), PDMS, and silicone rubber. Stiffness discrimination was achieved through active finger pressing motions. During the downward bending motion of the robotic fingers, the sensors continuously monitored changes in *∆S_z_*. Contact with the target object was confirmed once *∆S_z_* exceeded a preset threshold. After contact, the finger performed short‐stroke reciprocating oscillations at a fixed angle (±10°). Throughout this process, the peak *∆S_z_* values were measured and used to distinguish the local elastic stiffness of the different materials. Notably, the measured *∆S_z_* values confirmed the expected stiffness gradient, with sponge exhibiting the softest response and silicone the hardest (Figure 5c; Figure S14). As shown in Figure 5c, experimental results showed significant differences in *∆S_z_* peak responses among the materials, with clearly defined classification boundaries, demonstrating that this method can accurately and non‐destructively distinguish the flexibility differences across a wide range of representative materials.

For active grasping, we implemented a hierarchical perception and independent control strategy. The 16 sensors are divided into an active control layer (fingertip/finger‐pad) and an auxiliary monitoring layer (finger‐root/palm). In the control loop, the fingertip and finger‐pad sensors of each finger are fused via vector summation of synchronized 3D forces, yielding total *F_n_* and *F_s_* for each finger. This expands the effective sensing area and enables continuous feedback during contact shifts. Palm and finger‐root sensors are excluded from the active force feedback loop, as the palm acts as a passive support and the finger root is seldom a primary contact point. The hand thus operates as a coordinated set of two‐fingered grippers, with each finger independently adjusting its joints based on local fused feedback. To demonstrate the active grasping capability of the dexterous hand, four plastic bottles with identical external characteristics but incremental liquid masses ranging from 100 to 400 mL were used as test objects, as shown in Figure 5d. During trials, each bottle was placed on a retractable platform. At a moment after the contact was established, the platform was abruptly withdrawn to assess grasp stability.

In the baseline configuration, grasping relied solely on *F_z_* signals, with the clamping force threshold set between 0.1 N (to prevent slippage) and 0.4 N (to avoid excessive deformation). Under this setup, the 100 mL bottle remained in place but exhibited persistent instability, characterized by continuous finger repositioning and pronounced oscillations in the index finger's normal force (Figure 5e). For bottles containing 200–400 mL of liquid, significant downward slippage occurred during grasping, ultimately leading to object drops (Figure 5e,g; Movie S6). Analysis revealed that these failures stemmed primarily from the strategy's dependence on normal force feedback alone, which prevented early detection of incipient slippage. Moreover, as the bottle slipped, intermittent contact with the fingers produced transient spikes in the measured *F_z_* signal. For instance, as shown in Figure 5i, the 400 mL bottle exhibited such spikes between 19.5 and 20 s. These spikes risked being misinterpreted as excessive grip force, triggering an unintended reduction in clamping force and further accelerating object loss.

In contrast, the experimental group implemented a dynamic force adjustment mechanism leveraging 3D force information. This strategy employed concurrent *F_s_* and *F_n_* measurements to compute the force ratio *r*, with a stable operational regime was defined at *r* ∈ [0.4,0.65]. Upon detecting r deviations beyond thresholds, the controller immediately modulated finger joint kinematics. Results demonstrated significant improvement: all test masses (100‐400 mL) maintained stable grasps after support removal (Figure 5f; Movie S6). In the 400 mL case (Figure 5h), the platform was removed at approximately 20  s, inducing simultaneous r‐spikes (*r* > 0.7) on multiple fingers and enabling immediate slippage identification. Within 500 ms, the bottle's sliding trend was effectively halted. Subsequently, over nearly 1–2 s, the controller fine‐tuned the grip force of each finger to an appropriate level, restoring the force ratio *r* to the stable range (Figure 5j). The experiment clearly demonstrates that relying solely on normal force feedback has significant limitations when handling objects with substantial mass variations or slip risks. In contrast, integrating 3D force information and introducing a dynamic adjustment mechanism based on the force ratio r effectively enhances grasp reliability, and adaptability, providing crucial support for dexterous manipulation in complex environments.

To further evaluate generalizability, the system was tested on objects with diverse morphologies: pen, pine cone, balloon, and game controller (Figure 5k–n). Stable grasps were consistently achieved across all categories (Movie S7). Moreover, a dynamic robustness test was performed using the game controller to assess performance under contact drift (Figure S15 and Movie S7). Following a stable initial grasp, an external disturbance tilted the object, causing a shift in the contact interface. The system immediately detected the variations in *F_s_* and *F_n_*, triggering each finger to autonomously modulate its joint configuration. This adaptive response successfully restored a stable force ratio and effectively halted slippage. Furthermore, sequential grasping of objects with property differences (ping‐pong ball and balloon) was successfully executed (Figure 5o; Figure S16 and Movie S7). These results robustly validate the proposed 3D force‐feedback strategy as exhibiting outstanding generality and robustness in complex real‐world scenarios.

## 3 Conclusions

Dexterous hands serve as the primary interface for robot‐environment interaction, requiring 3D force sensing for stable and reliable manipulation in complex environments. Magnetic tactile sensors achieve effective 3D force perception by leveraging centripetal magnetization designs and processing multi‐axis magnetic signals. Notably, the force sensing accuracy of a magnetic tactile sensor is critically constrained by the fabrication precision of its magnetic film. Consequently, integrating these sensors into dexterous fingertips presents a pivotal manufacturing challenge: achieving high‐precision processing of miniaturized magnetic films with centripetal magnetization. To overcome this, our study proposed a novel LAFM process. The method introduced laser‐etched grooves to reduce local stiffness for precise folding, followed by magnetization to create precise centripetal structures. The fabricated miniature magnetic units (5 × 5 mm^2^) exhibited excellent agreement with theoretical models in all magnetic induction components (*B_x_*, *B_y_*, *B_z_*), with RMSE below 5 µT, confirming the superiority of this process. Based on this technology, we developed a compact 3D force sensor capable of high‐precision displacement decoupling with sub‐millimeter resolution (0.01 mm). The sensor achieves reliable force measurement within ranges of 4.5 N (normal) and 0.45 N (tangential), with corresponding resolutions of 3 and 9 mN. It demonstrates a fast dynamic response time of 34 ms, and maintains an excellent stability over 2500 loading cycles (<1% deviation). The LAFM sensors were deployed on multiple robotic platforms, demonstrating a progression from adaptive grasping to advanced perception. On a mobile manipulator, it ensured a secure hold on a fragile cherry tomato while traversing obstacles. Progressing to more complex tasks, a dexterous hand equipped with sixteen units provided comprehensive force feedback. This enabled non‐destructive stiffness discrimination across six representative materials and stable grasping of diverse objects. Furthermore, the full 3D force feedback proved critical, significantly outperforming systems reliant on normal force alone by reliably handling objects of variable mass (e.g., 100–400 mL of liquid) amid sudden disturbances.

However, considerable opportunities for improvement remain in future applications. On the one hand, it is essential to further increase the integration density of the sensors. By introducing femtosecond laser and advanced magnetization techniques, it is expected to enable the monolithic production of large‐area magnetic film arrays. Such progress will also facilitate seamless integration onto complex curved surfaces, thereby meeting the diverse tactile sensing requirements of different robotic morphologies. On the other hand, there is ample potential to expand the functional dimensions of the sensing system. Beyond force measurement, the systems functionality can be extended to incorporate capabilities for position detection and shape recognition. This multimodal integration could enable a more comprehensive understanding of the external environment. Additionally, given the temperature‐sensitive nature of Hall sensors, development efforts will focus on integrating temperature sensors with real‐time compensation algorithms. This integration is crucial for enhanced robustness and environmental adaptability. In the future, the LAFM‐based tactile sensors are anticipated to serve as core sensing elements in next‐generation robots. They will not only endow robots with more stable and fine‐grained manipulation capabilities but also promote progress toward autonomous execution of increasingly complex and diverse tasks.

## 4 Methods

### 4.1 Fabrication and Magnetization of Magnetic Films

The preparation process of the magnetic film is as follows. First, the following raw materials are weighed and mixed: SE 1700 base (DOWSIL, 11.71 wt%), SE 1700 catalyst (DOWSIL, 1.17 wt%), Ecoflex 00–30 Part B (Shanghai Zhixin Corp., 21.78 wt%), fumed silica nanoparticles (Aladdin Biochemical Technology Corp., 2.72 wt%), and NdFeB magnetic particles (Jianghuai Ciye Corp., 62.62 wt%) [46]. The addition of fumed silica nanoparticles is intended to adjust the rheological properties of the slurry. They are placed in a centrifugal mixer and stirred at 2000 r/min for 3 min to ensure thorough mixing, followed by defoaming at 2000 r/min for 2 min. The mixed slurry is then cast onto a glass plate coated with a release film. A spacer of a certain thickness (e.g., an aluminum plate) is used to control the thickness of the magnetic film. Another glass plate is placed on top and secured with clamps. The assembly is placed in an oven and cured at 100°C for 60 min. After curing, the formed magnetic film is peeled off. The desired crease grooves are processed using a carbon dioxide laser cutter, and the film is folded with the aid of auxiliary jigs. Finally, the film is magnetized in a high‐intensity magnetic field to achieve centripetal magnetization.

### 4.2 Fabrication Process of Miniaturized 3D Force Sensor

As shown in Figure S1, double‐sided adhesive is applied to the bottom of the printed mold to secure the magnetized magnetic film. Subsequently, a mixture of Ecoflex 00–30 parts A and B (at a 1:1 ratio) is poured into the mold. To modify the hardness of this layer, a controlled amount of PDMS or silicone oil can be incorporated into the mixture. After curing, this elastomeric layer forms the flexible sensing region. The MLX90393 Hall sensor is then encapsulated using a rigid protective material or mounted via a custom‐designed mechanical structure. Finally, the flexible sensing layer and the sensor module are bonded together to complete the assembly of the integrated functional structure.

### 4.3 Circuit Design

The tactile sensing system of the dexterous hand consists of an array of 16 Hall sensors mounted on a flexible PCB. To avoid address conflicts, the system assigns unique addresses to all 16 sensors by adjusting the A0 and A1 address pins as well as model configurations. Each sensor can perform real‐time measurements of the 3D magnetic field. This measurement data, when combined with input from a magnet, can be converted into 3D tactile stress information. In terms of hardware architecture, the system employs the STM32H7 as the main controller. The system makes use of its four independent I^2^C interfaces to connect the 16 sensors in groups, with each interface managing 4 sensors. This design not only prevents bus communication bottlenecks but also enables millisecond‐level full‐array data refresh, ensuring both the real‐time performance and stability of tactile signal acquisition.

## Author Contributions

Y.H. led the development of the concepts, designed the experiments. Y.H. and H.D. processed the data, and interpreted results. H.D. and C.Z construct the theoretical model. A.D. and X.Z. partially participated in the experiments. Y.H. drew the figures, and wrote the paper. C.Z., H.D., and D.T. conceived the idea and revised the manuscript. P.Z., C.Z., and H.D. provided funding support. All authors participated in the discussion of this project.

## Conflicts of Interest

The authors declare no conflicts of interest.

## Supporting information




**Supporting File**: advs74687‐sup‐0001‐SuppMat.docx.

**Supporting File**: advs74687‐sup‐0002‐MovieS1.mp4.

**Supporting File**: advs74687‐sup‐0003‐MovieS2.mp4.

**Supporting File**: advs74687‐sup‐0004‐MovieS3.mp4.

**Supporting File**: advs74687‐sup‐0005‐MovieS4.mp4.

**Supporting File**: advs74687‐sup‐0006‐MovieS5.mp4.

**Supporting File**: advs74687‐sup‐0007‐MovieS6.mp4.

**Supporting File**: advs74687‐sup‐0008‐MovieS7.mp4.

## Data Availability

The data that support the findings of this study are available from the corresponding author upon reasonable request.
