# Supplementary material for: Miniaturized 3D Magnetic Force Sensor via Laser‐Assisted Folding and Magnetization for Enhanced Robotic Dexterity
Source: Adv Sci (Weinh). 2026 Mar 27;13(29):e24321. doi: 10.1002/advs.202524321 (PMC13205680; doi:10.1002/advs.202524321)
Supplement: Supplementary file 1 — Supporting File: advs74687‐sup‐0001‐SuppMat.docx. [file ADVS-13-e24321-s007.docx]

**Supplementary Materials for “Miniaturized 3D Magnetic Force Sensor via Laser-Assisted Folding and Magnetization for Enhanced Robotic Dexterity”**

Yujie Huang^1,2^, Huangzhe Dai^1,2^, Chengqian Zhang^1,2^*, Daofan Tang^1,2^, Xinxin Zhang^1^, Haonan Sun^1,2^, Annan Ding^1,2^, Xuebin Ni^1^, Yizhi Zhang^1^, Chengfeng Pan, Peng Zhao^1,2^*

(^1^ The State Key Laboratory of Fluid Power and Mechatronic Systems, College of Mechanical Engineering, Zhejiang University, Hangzhou 310027, China

^2^ Zhejiang Key Laboratory of Additive Manufacturing Technology and Equipment, School of Mechanical Engineering, Zhejiang University, Hangzhou 310058, China

^*^ Corresponding Author Email: [zhangcq@zju.edu.cn](mailto:zhangcq@zju.edu.cn); [pengzhao@zju.edu.cn](mailto:pengzhao@zju.edu.cn) )

**The PDF file includes:**

Supplementary Table 1. Comparison of state-of-the-art magnetic tactile sensors, measurement dimensions, decoupling methods and unit size.

Supplementary Table 2. Parameters for mechanical deformation simulation of structures with and without grooves.

Supplementary Table 3. Parameters for magnetic field simulation of magnetic films with and without grooves.

Supplementary Figure 1. Fabrication process of miniaturized 3D force sensor.

Supplementary Figure 2. Influence of processing parameters on the morphology of laser-etched grooves.

Supplementary Figure 3. Effect of groove geometry on film deformation.

Supplementary Figure 4. Repeatability of groove fabrication under optimized parameters.

Supplementary Figure 5. Simulation of magnetic field underneath the magnetic film with and without groove structures.

Supplementary Figure 6. Simulation of spatial 3D magnetic field distribution beneath the magnetic film with groove structures.

Supplementary Figure 7. Decoupling performance of 3D displacement for grooved magnetic films of different sizes.

Supplementary Figure 8. Signal stability evaluation of the sensor in air versus underwater conditions.

Supplementary Figure 9. Comparative experiments of grasping strategies for cups of varying mass.

Supplementary Figure 10. Real-time 3D force and force ratio data for closed-loop egg grasping under disturbance.

Supplementary Figure 11. 3D force response of a cherry tomato from non-destructive grasping to rupture failure.

Supplementary Figure 12. Continuous damage-free grasping of five lightweight fragile objects.

Supplementary Figure 13. Simulation analysis of magnetic crosstalk between adjacent sensor units.

Supplementary Figure 14. Hardness measurement of six typical materials using a hardness tester.

Supplementary Figure 15. Adaptive finger reconfiguration in response to contact drift.

Supplementary Figure 16. Sequential grasping of diverse objects and real-time UI feedback.

Supplementary Reference

**Supplementary Table 2.** Comparison of state-of-the-art magnetic tactile sensors, measurement dimensions, decoupling methods and unit size.

| Magnetic tactile sensor type | Force output | | | | | Sensor feature | |
| --- | --- | --- | --- | --- | --- | --- | --- |
|  | Decoupling method | Normal force | Shear force | Decoupling  dimension | Resolution | Magnetization method | Unit size |
| 3D magnetic tactile sensors with high sensing accuracy^[1]^ | Data fitting | Yes | Yes | Three dimensions | Not reported | Rigid cylindrical permanent magnet | 3mm (26mm) |
| Flexible wide-range multidimensional force sensors^[2]^ | Data fitting | Yes | Yes | Two dimensions | Not reported | Rigid cylindrical permanent magnet | 4mm (15mm) |
| 3-D force-temperature tactile sensor^[3]^ | Data fitting | Yes | Yes | Three dimensions | Not reported | Unidirectional magnetization | 8mm |
| Tactile sensor based on magnetic sensing^[4]^ | Data fitting | Yes | Yes | Two dimensions | 2mN | Unidirectional magnetization | 8mm |
| Soft magnetic skin for super-resolution tactile sensing^[5]^ | Formula derivation | Yes | Yes | Two dimensions | 10mN | Sinusoidal magnetization (curling) | 6mm |
| Split-type magnetic soft tactile sensor^[6]^ | Formula derivation | Yes | Yes | Three dimensions | 13mN | Centripetal magnetization (folding) | 20mm |
| Soft skin with self-decoupled three-axis force-sensing taxels^[7]^ | Formula derivation | Yes | Yes | Three dimensions | Not reported | Sinusoidal magnetization (curling and overlaying) | 8mm |
| Soft tactile unit with 3D force and temperature decoupling ability^[8]^ | Formula derivation | Yes | Yes | Three dimensions | 10mN | Sinusoidal magnetization (curling and splicing) | 12mm |
| Sandwich Miura-Ori enabled large area, super resolution tactile skin^[9]^ | Formula derivation | Yes | Yes | Three dimensions | Not reported | Centripetal magnetization (splicing) | 13mm |
| **This work** | **Formula derivation** | **Yes** | **Yes** | **Three dimensions** | **3mN** | **Centripetal magnetization (LAFM)** | **5mm** |

**Supplementary Table 2.** Parameters for mechanical deformation simulation of structures with and without grooves.

| Parameter | Value |
| --- | --- |
| Density ($\rho$) | 2.5 ×10^3^ [$kg/\mathrm{mm}^{3}$] |
| Young’s modulus (E) | 0.8 [Mpa] |
| Possion’s ratio ($\nu$) | 0.49 |

**Supplementary Table 3.** Parameters for magnetic field simulation of magnetic films with and without grooves.

| Parameter | Value |
| --- | --- |
| Side length (l) | 5 [mm] |
| Thickness (d) | 0.25 [mm] |
| Etched depth ($d_{1}$) | 0.2 [mm] |
| Etched width (dl) | 0.1 [mm] |
| Remanence ($m_{0}$) | 0.244 [T] |
| Wavelength ($\lambda$) | 5 [mm] |
| Range of the study | z = -5.75 - 5 mm |


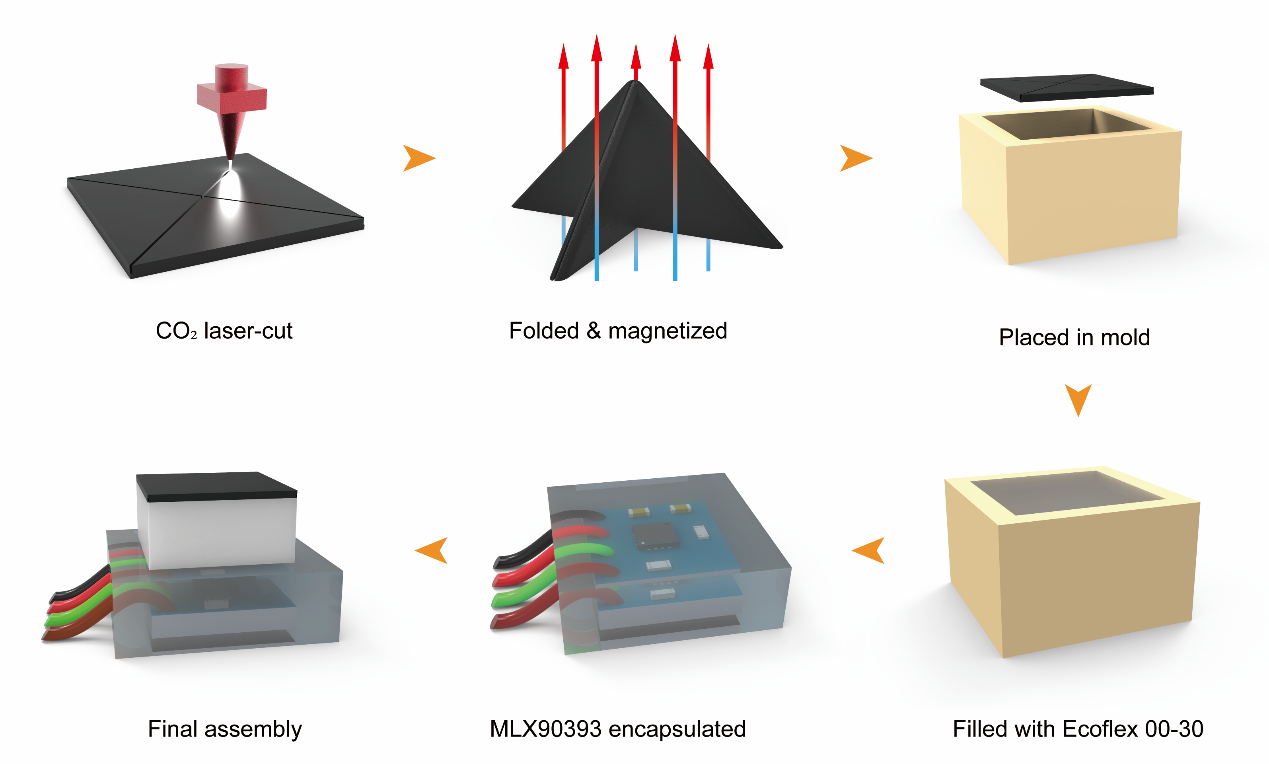


**Supplementary Figure 1.** Fabrication process of miniaturized 3D force sensor.


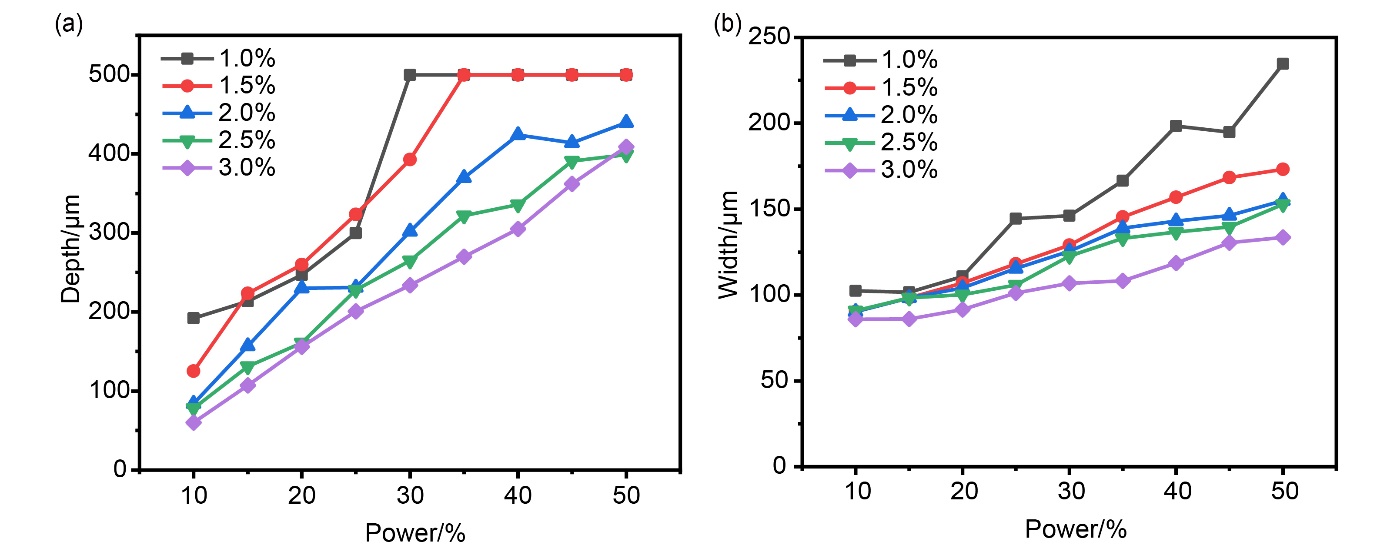


**Supplementary Figure 2.** Influence of processing parameters on laser-etched groove geometry. (a) Variation of groove depth with laser power and scan speed. (b) Variation of groove width with laser power and scan speed.


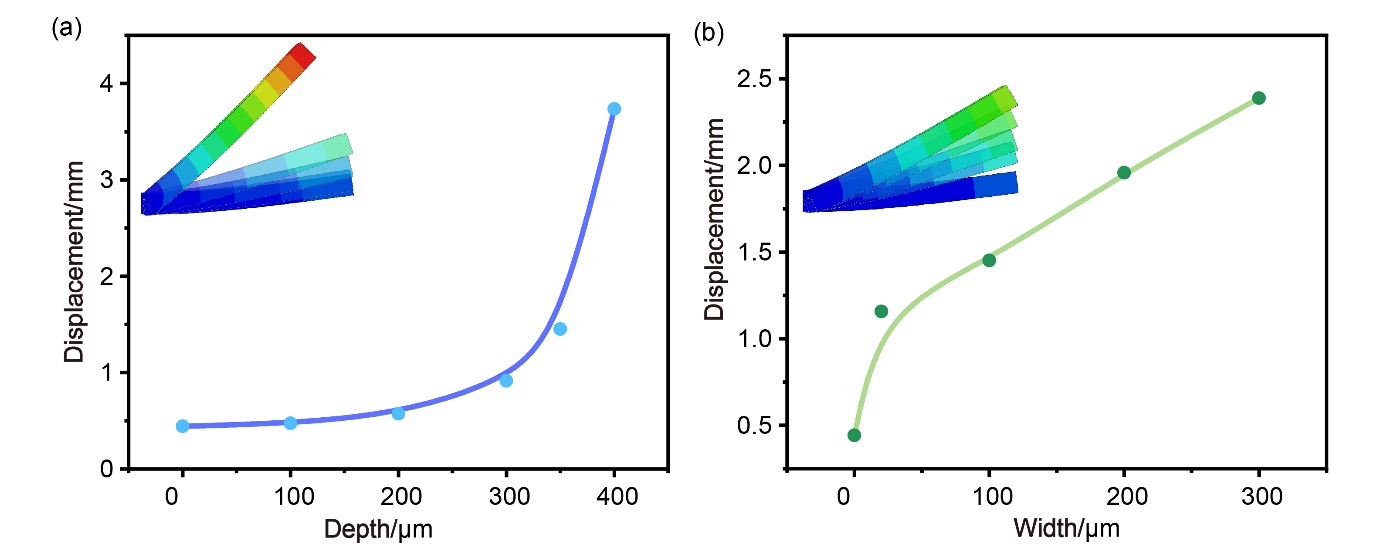


**Supplementary Figure 3.** Effect of groove geometry on film deformation. (a) Effect of groove depth. (b) Effect of groove width.


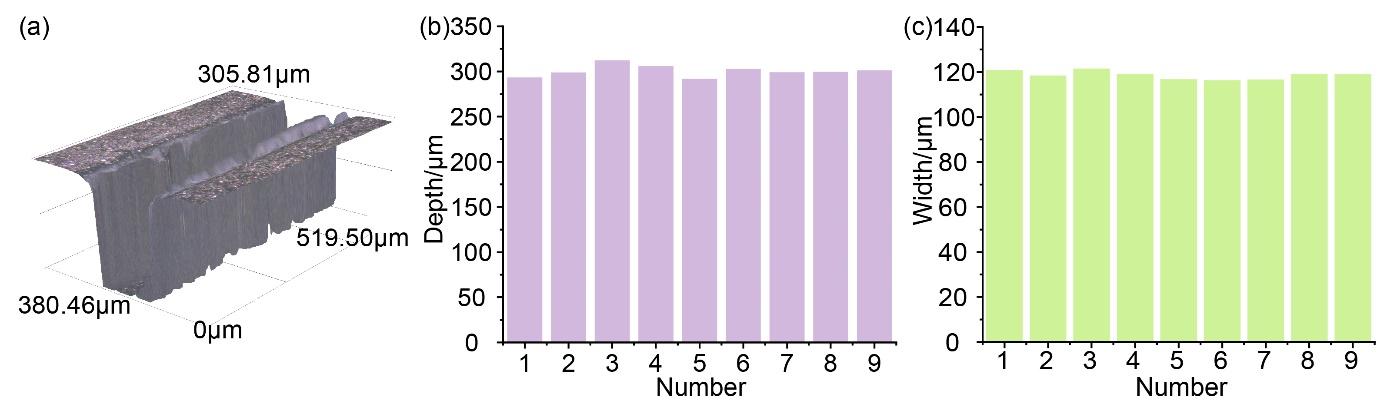


**Supplementary Figure 4.** Repeatability of groove fabrication under optimized parameters. (a) 3D topography of a representative laser-etched groove. (b) Measured groove depth across nine samples. (c) Measured groove width across nine samples.


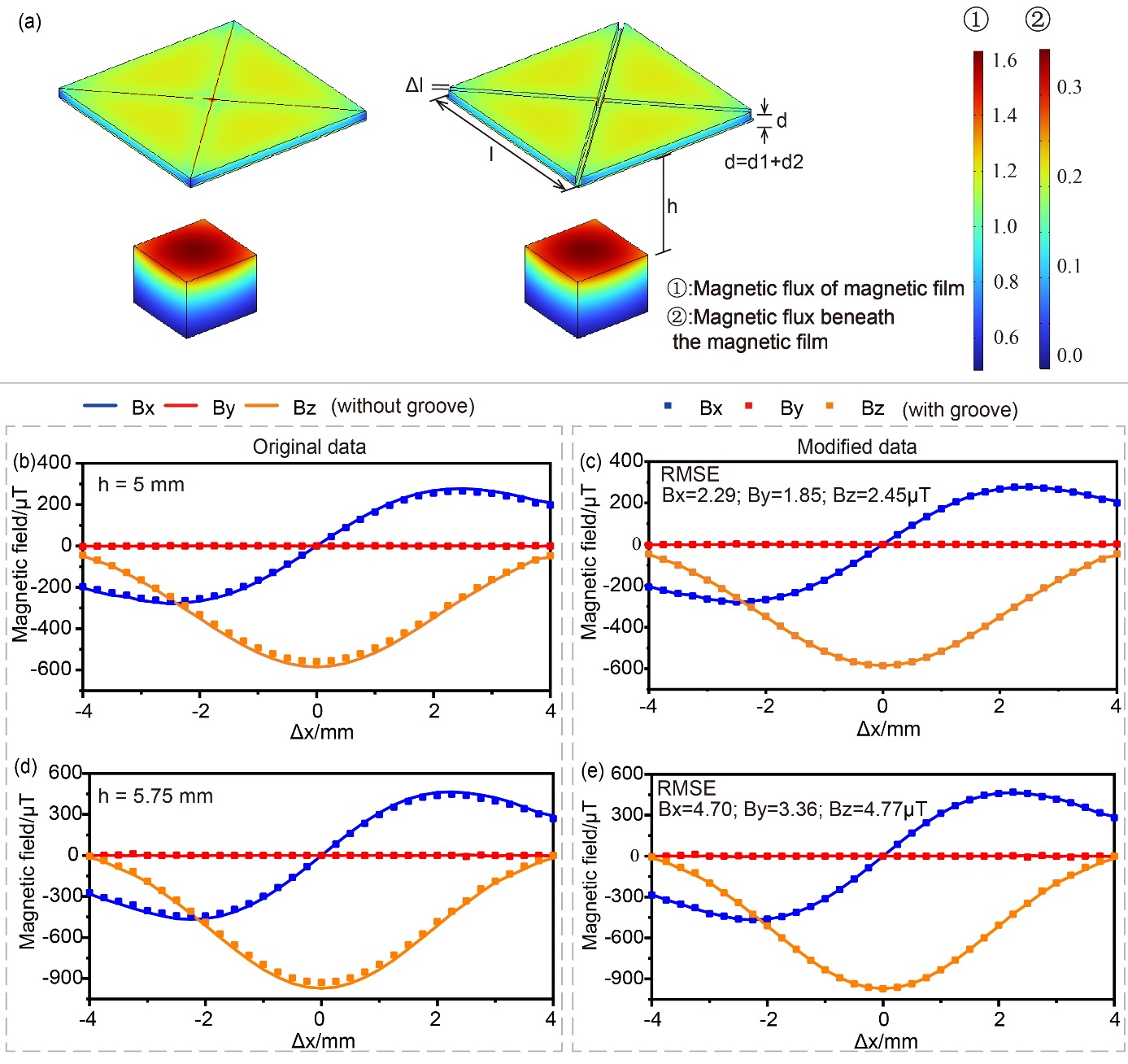


**Supplementary Figure 5.** Simulation of magnetic field underneath the magnetic film with and without groove structures. (a) Experimental data visualization and key parameter settings. (b) Comparison of magnetic field distribution along the *x*-axis with and without grooves (h = 5 mm). (c) Comparison of *a_0_*-corrected magnetic field distribution along the *x*-axis (h = 5 mm, *a_0_* = 1.04). (d) Comparison of magnetic field distribution along the *x*-axis with and without grooves (h = 5.75 mm). (e) Comparison of *a_0_*-corrected magnetic field distribution along the x-axis (h = 5.75 mm, *a_0_* = 1.04).


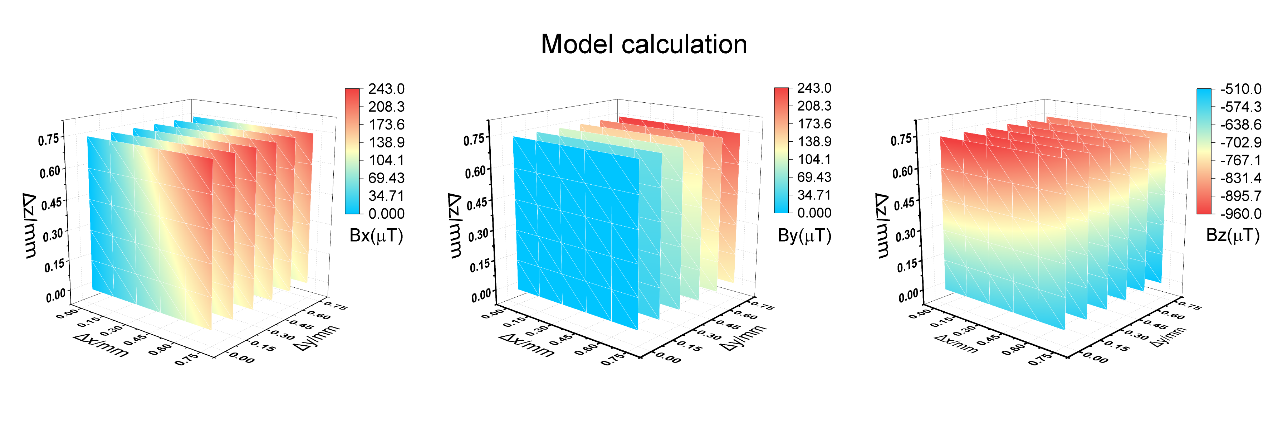


**Supplementary Figure 6.** Simulation of spatial 3D magnetic field distribution beneath the magnetic film with groove structures.


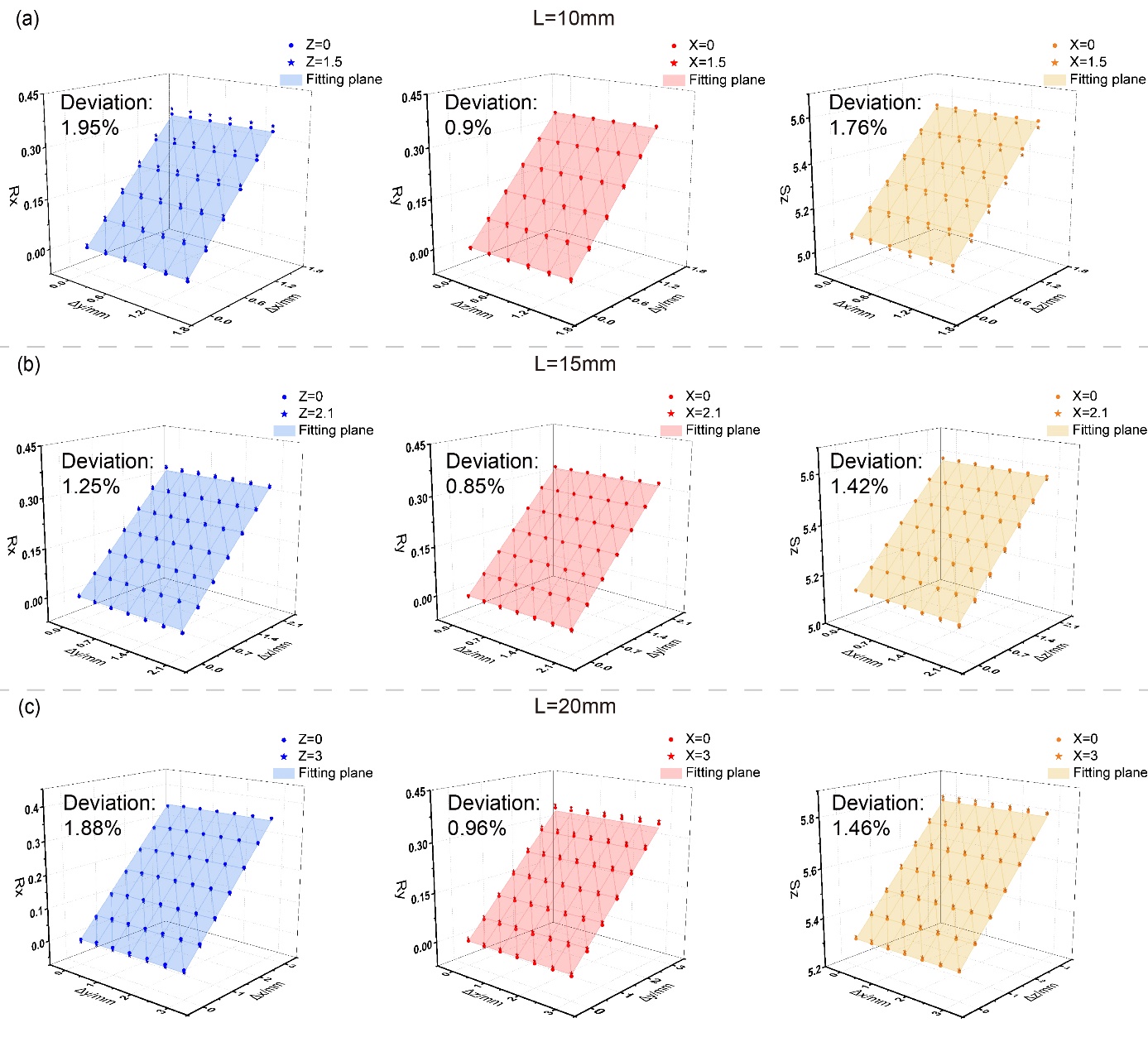


**Supplementary Figure 7.** Decoupling performance of 3D displacement for grooved magnetic films of different sizes. (a) With a film side length of 10 mm. (b) With a film side length of 15 mm. (c) With a film side length of 20 mm.


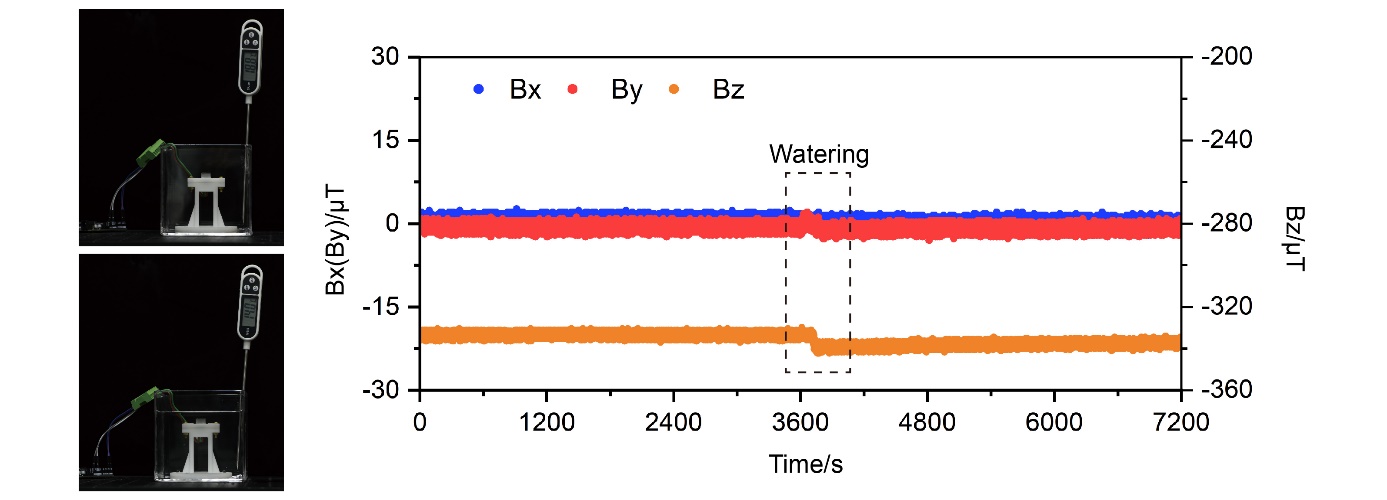


**Supplementary Figure 8.** Signal stability evaluation of the sensor in air versus underwater conditions.


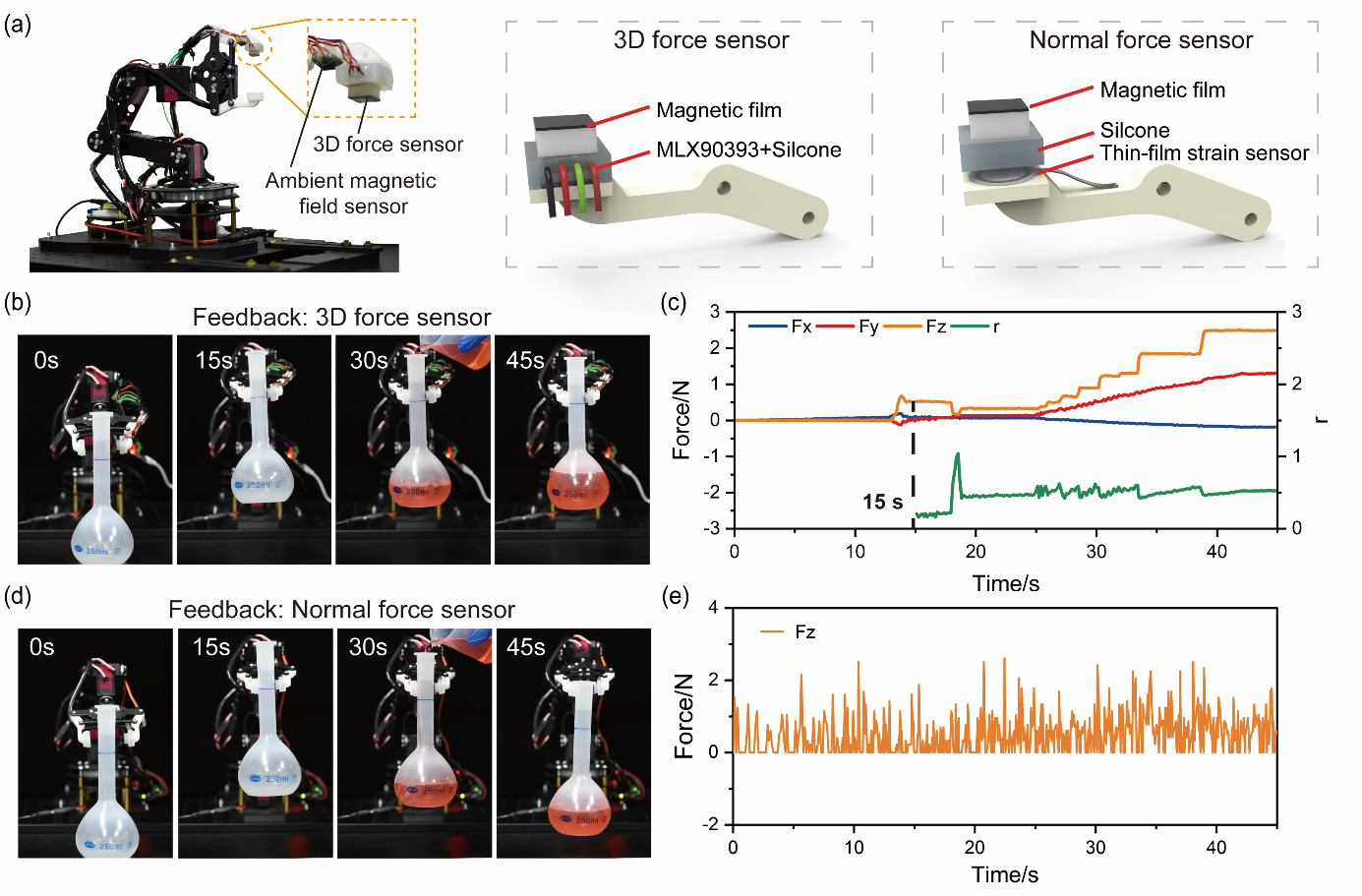


**Supplementary Figure 9.** Comparative experiments of grasping strategies for cups of varying mass. (a) Experimental setup and sensor configuration on the robotic arm. (b) Robotic grasping process based on 3D force feedback. (c) 3D force and force ratio *r* curves during grasping with 3D force feedback. (d) Robotic grasping process based on normal force feedback. (e) *F_z_* curve during grasping with normal force feedback.


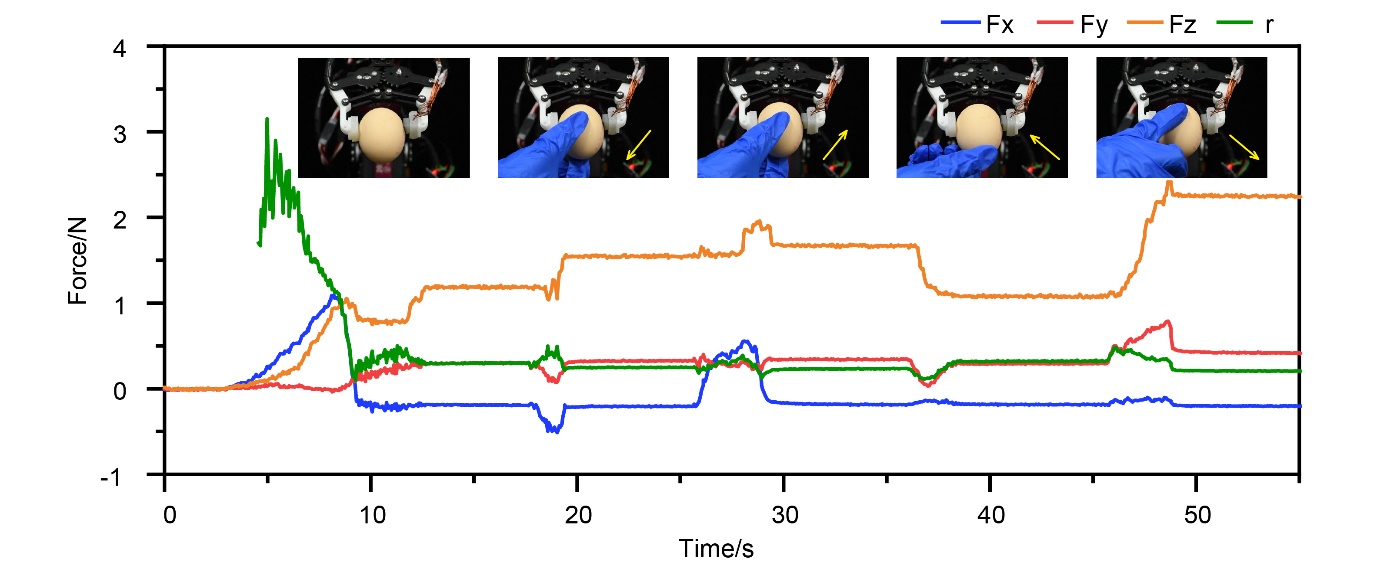


**Supplementary Figure 10.** Real-time 3D force and force ratio data for closed-loop egg grasping under disturbance.


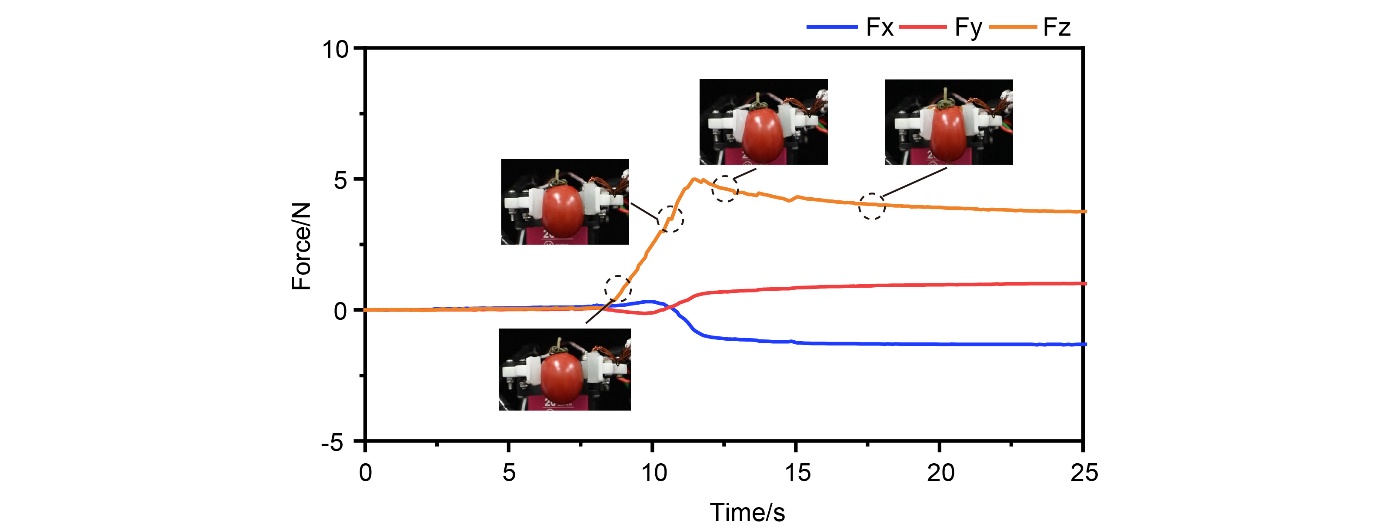


**Supplementary Figure 11.** 3D force response of a cherry tomato from non-destructive grasping to rupture failure.


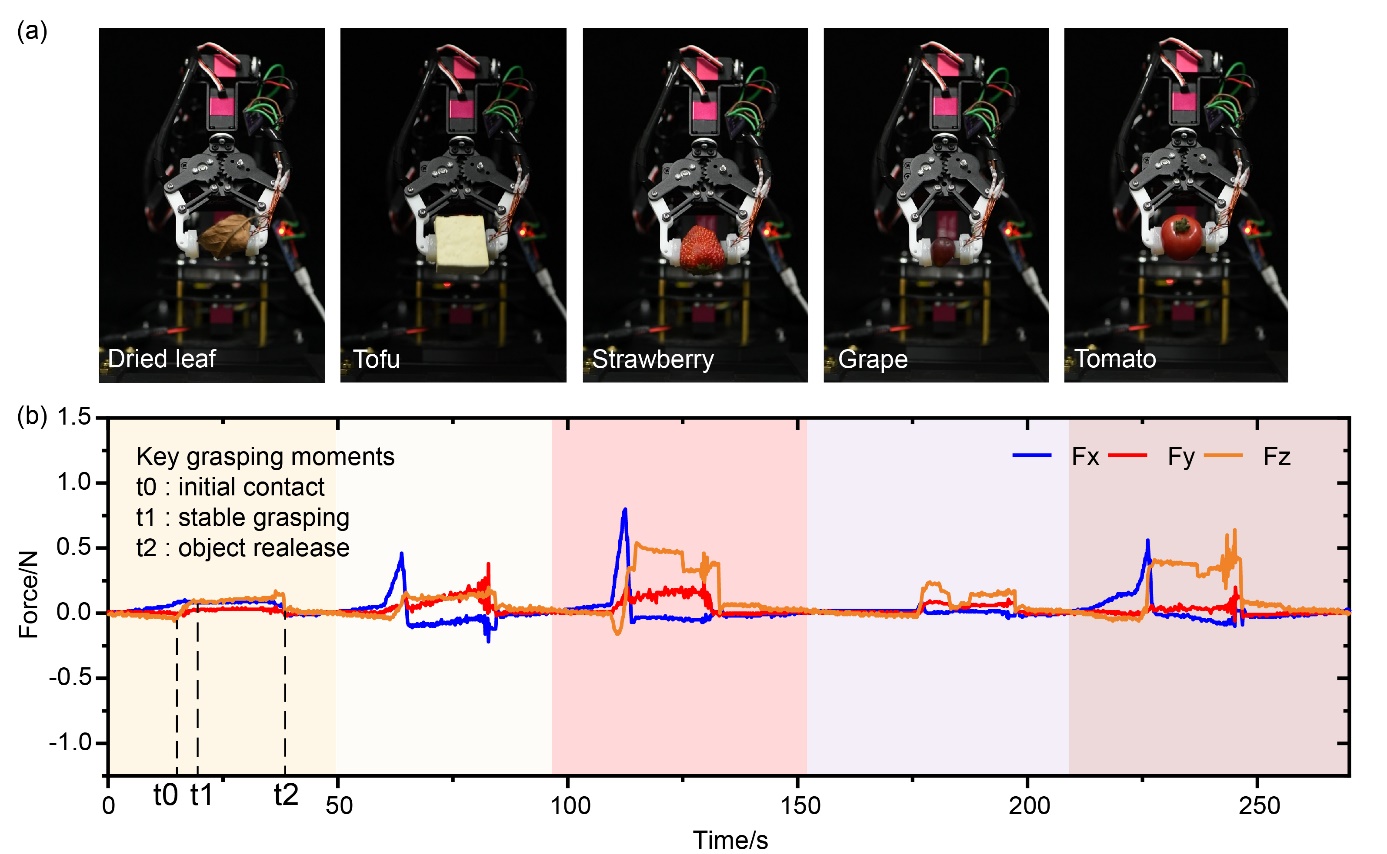


**Supplementary Figure 12.** Continuous damage-free grasping of five lightweight fragile objects. (a) Experimental snapshots of successful grasps. (b) Corresponding real-time 3D force profiles.


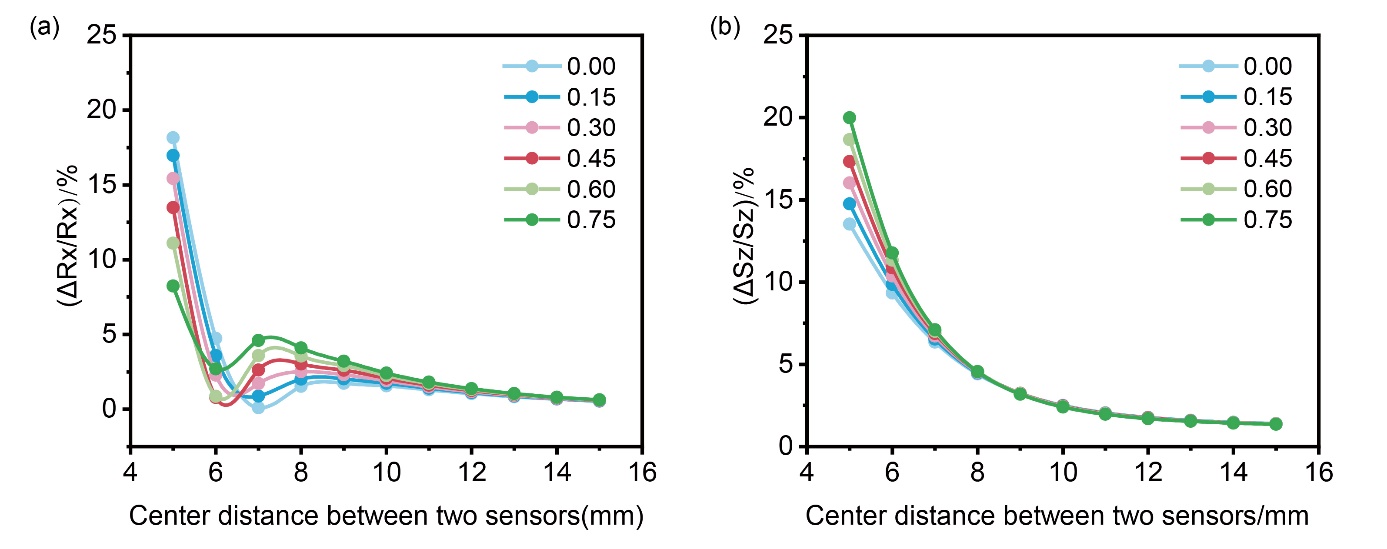


**Supplementary Figure 13.** Simulation analysis of magnetic crosstalk between adjacent sensor units. (a) Influence of inter-unit spacing on the relative deviation of decoupling coefficient *R_x_*; (b) Influence of inter-unit spacing on the relative deviation of decoupling coefficient *S_z_*.


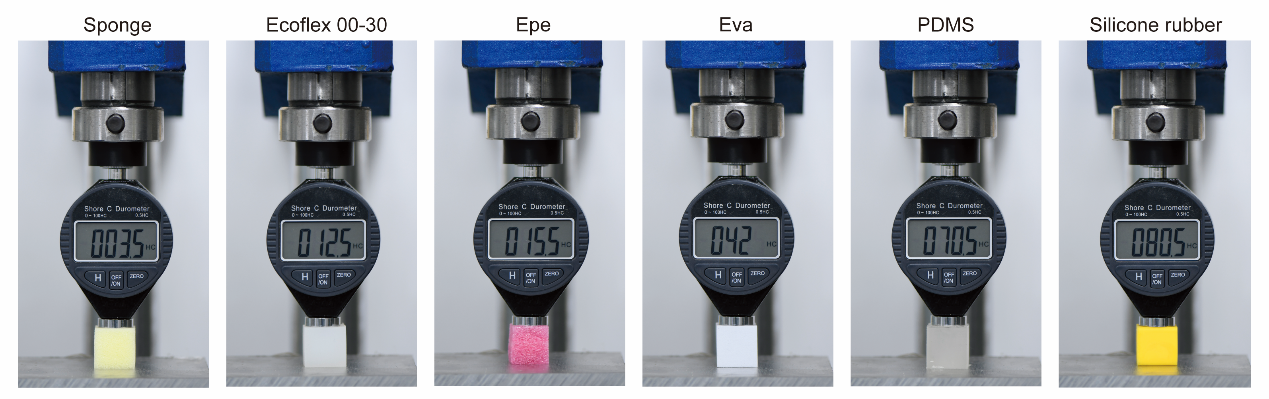


**Supplementary Figure 14.** Hardness measurement of six typical materials using a hardness tester.


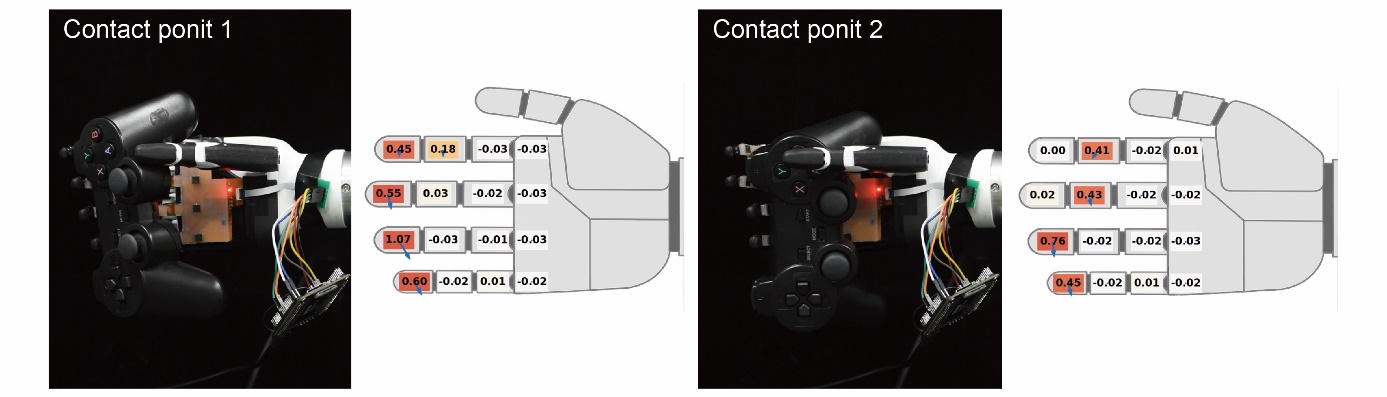


**Supplementary Figure 15.** Adaptive finger reconfiguration in response to contact drift.


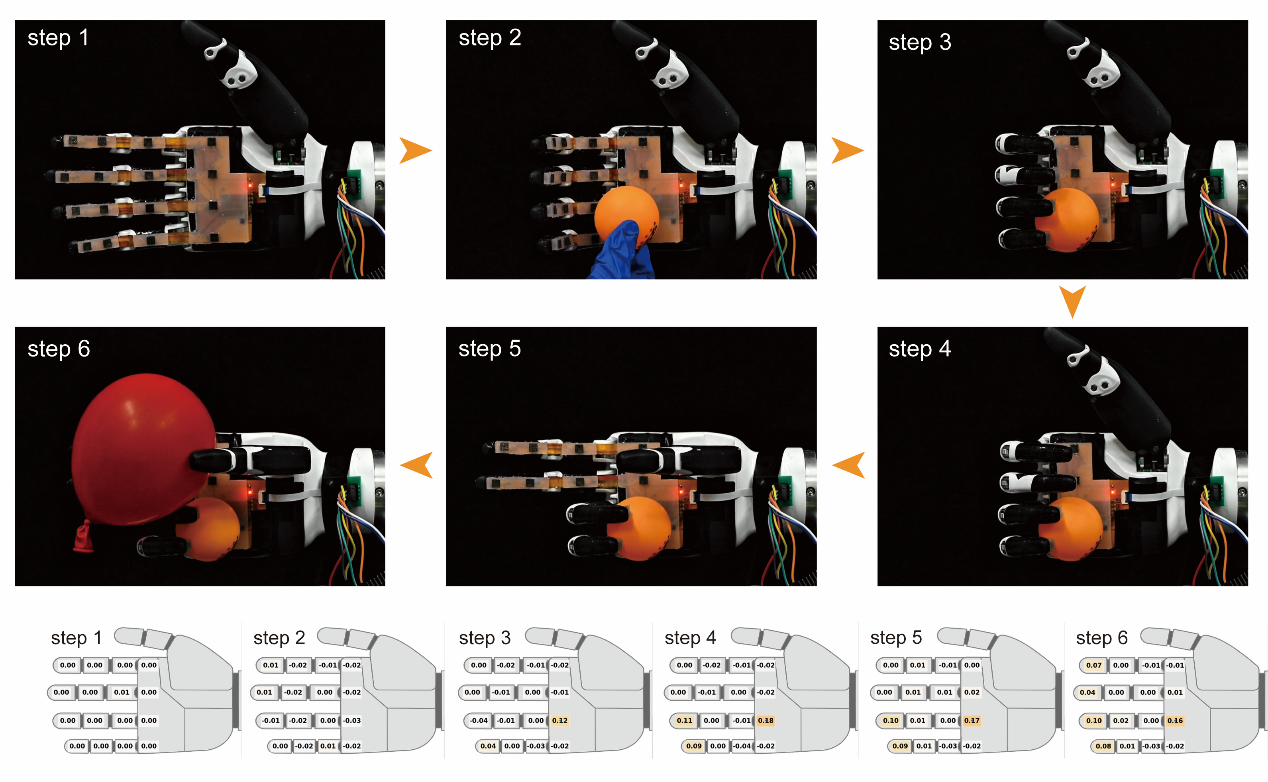


**Supplementary Figure 16.** Sequential grasping of diverse objects and real-time UI feedback.

**Reference**

[1] X. Hu, H. Zhu, R. Chen, S. Hu, Z. Jia, H. Yu, S. Qu, *Advanced intelligent systems* **2023**, *5*, 2200291.

[2] J. Zhang, X. Hou, S. Qian, J. Huo, M. Yuan, Z. Duan, X. Song, H. Wu, S. Shi, W. Geng, J. Mu, J. He, X. Chou, *Microsystems & nanoengineering* **2024**, *10*, 64.

[3] S. Zuo, L. Weng, X. Ji, L. Hao, X. Du, B. Cui, *Measurement: journal of the International Measurement Confederation* **2025**, *256*, 118534.

[4] J. Li, H. Qin, Z. Song, L. Hou, H. Li, *IEEE transactions on instrumentation and measurement* **2024**, *73*, 1.

[5] Y. Yan, Z. Hu, Z. Yang, W. Yuan, C. Song, J. Pan, Y. Shen, *Science robotics* **2021**, *6*, eabc8801.

[6] H. Dai, C. Zhang, C. Pan, H. Hu, K. Ji, H. Sun, C. Lyu, D. Tang, T. Li, J. Fu, P. Zhao, *Advanced materials* **2024**, *36*, 2310145.

[7] Y. Yan, A. Zermane, J. Pan, A. Kheddar, *Nature machine intelligence* **2024**, *6*, 1284.

[8] X. Yang, H. Ren, D. Guo, Z. Ling, T. Zhang, G. Li, Y. Tang, H. Zhao, J. Wang, H. Chang, T. Gao, J. Dong, N. Wu, Y. Shen, *Engineering* **2025**, *55*, 96.

[9] Q. Xu, Z. Yang, Z. Wang, R. Wang, B. Zhang, Y. Cheung, R. Jiao, F. Shi, W. Hong, H. Yu, *Advanced science* **2025**, *12*, 2414580.
